# Clustered micronodules as predominant manifestation on CT: A sign of active but indolently evolving pulmonary tuberculosis

**DOI:** 10.1371/journal.pone.0231537

**Published:** 2020-04-17

**Authors:** Jung Hee Hong, Soon Ho Yoon, Jin Mo Goo, Jae-Joon Yim, Yoon Kyung Jeon

**Affiliations:** 1 Department of Radiology, Seoul National University College of Medicine, Seoul National University Hospital, Seoul, Korea; 2 Division of Pulmonary and Critical Care Medicine, Department of Internal Medicine, Seoul National University College of Medicine, Seoul, Korea; 3 Department of Pathology, Seoul National University College of Medicine, Seoul National University Hospital, Seoul, Korea; Vanderbilt University Medical Center, UNITED STATES

## Abstract

**Objective:**

To investigate the prevalence, patient characteristics, and natural history of clustered micronodules (CMs) in active pulmonary tuberculosis.

**Materials and methods:**

From January 2013 through July 2018, 833 consecutive patients with bacteriologically or polymerase chain reaction–proven active pulmonary tuberculosis were retrospectively evaluated. CMs were defined as a localized aggregation of multiple dense discrete micronodules, which primarily distributed around small airways distal to the level of the segmental bronchus: small airways surrounded by CMs maintained luminal patency and the CMs might coalesce into a larger nodule. The patients were dichotomized according to whether the predominant computed tomography (CT) abnormalities were CMs. We analyzed radiologic and pathologic findings in patients whose predominant diagnostic CT abnormalities were CMs, along with those of incidental pre-diagnostic CT scans, if available. Chi-square, McNemar, Student *t*-test and Wilcoxon-signed rank test were performed.

**Results:**

CMs were the predominant CT abnormality in 2.6% of the patients (22/833, 95% CI, 1.8–4.0%) with less sputum smear-positivity (4.8% vs 31.0%; *p* = .010) and a similar proportion of immunocompromised status (40.9% vs 46.0%; *p* = .637) than those without having CMs as the predominant CT abnormality. The time interval for minimal radiologic progression was 6.4 months. The extent of CMs increased with disease progression, frequently accompanied by consolidation and small airway wall thickening. Pathologically, smaller CMs were non-caseating granulomas confined to the peribronchiolar interstitium, whereas larger CMs were caseating granulomas involving lung parenchyma. Two of the five patients with a pre-diagnostic CT scan obtained more than 50 months pre-diagnosis showed an incipient stage of CMs, in which they were small peribronchiolar nodules.

**Conclusion:**

Active pulmonary tuberculosis manifested predominantly as CMs in 2.6% of patients, with scarce of acid-fast bacilli smear-positivity and no association with impaired host immunity. CMs indolently progressed, accompanied by consolidation and small airway wall thickening, and originated from small nodules.

## Introduction

*Mycobacterium tuberculosis* is the leading cause of death from a single infectious agent worldwide. Although the global incidence of tuberculosis (TB) has been declining steadily since 2003, 10.0 million new cases, with 1.6 million deaths occurred in 2017 [1]. When infected with TB in human, granuloma formation is a crucial host immune response to TB. TB granuloma is traditionally characterized by central caseation necrosis surrounded by a granulomatous inflammatory process. The typical radiologic findings of active pulmonary TB in an immunocompetent host corresponds to these characteristics. The typical findings include cavities, centrilobular micronodules, bronchial wall thickening, and tree-in-bud lesions on chest computed tomography (CT) images, and those findings represent caseation materials within or surrounding terminal or respiratory bronchioles and the alveolar duct, suggesting endobronchial spread of TB [2–4].

Active pulmonary TB can atypically manifest on radiologic examinations, especially in immunocompromised hosts [5]. One of the atypical manifestations is clustered micronodules (CMs) on chest CT images. The original definition of CMs in pulmonary TB was a large pulmonary nodular opacity composed of central coalescent micronodules, which can mimic the appearance of the ‘sarcoid galaxy’ sign [6–8]. However, the central coalescence was not mandatory for defining the CMs in other pieces of literature, and the CMs was literally defined as a simple cluster of micronodules regardless of central coalescence [9]. Furthermore, CMs pathologically represent not only caseating granulomas, but also non-caseating granulomas [3, 9]. Despite the varying radiologic and pathologic understandings of CMs, there is a paucity of studies on this unique form of active pulmonary TB.

The purpose of this study was to evaluate the prevalence, patient characteristics, and natural history of CMs in active pulmonary TB.

## Materials and methods

This study was approved by the institutional review board of Seoul National University Hospital, which waived informed consent (IRB No. H1806-084-951).

### Study population

From January 2013 through July 2018, we retrospectively reviewed the medical records of 3223 consecutive adult patients who were diagnosed with active pulmonary or extrapulmonary TB in a prospectively collected cohort at a single tertiary referral center. Among these patients, the study population was enrolled by applying the following criteria for eligibility: (a) bacteriological (sputum smear or culture) or polymerase chain reaction (PCR)–proven active pulmonary TB from respiratory specimens (sputum, bronchoalveolar lavage, open or percutaneous lung tissue biopsy); and (b) an available diagnostic CT scan obtained within 3 months of the bacteriological diagnosis before initiation of TB treatment. We excluded patients with nontuberculous mycobacterial lung disease and patients with a previous history of treatment for pulmonary TB.

Of the patients who met the above requirements, 833 (mean age, 62.3 years; 95% CI, 47.5–79.5 years; M:F, 62%:38%) were ultimately included in this study. A research assistant reviewed the electronic medical records of the patients to extract information on age, sex, smoking history, and the following risk factors that cause impaired host immune response to TB and facilitate TB progression: diabetes mellitus, chronic renal failure, previous history of malignancy, connective tissue disease, previous history of organ transplantation, and human immunodeficiency virus infection [10–12].

### Image acquisition

All patients underwent a chest CT scan with at least 16-channel or higher multi-detector CT scanners with or without contrast enhancement, with a fixed tube voltage of 120 kVp, with automatic exposure control, and an image slice thickness ranging from 1 to 5 mm. The majority of CT scans were obtained with one of the following six CT scanners: Aquilion ONE (Toshiba, Tokyo, Japan); Discovery CT750 HD (GE Healthcare, Milwaukee, WI, USA); iCT 256/Ingenuity CT (Philips, Amsterdam, the Netherlands); SOMATOM Definition Flash/Force (Siemens Medical System, Erlangen, Germany). The mean interval between the diagnostic CT exam and the initiation of TB treatment was 16.4 ± 19.7 days.

### Image assessment

**Fig 1:**
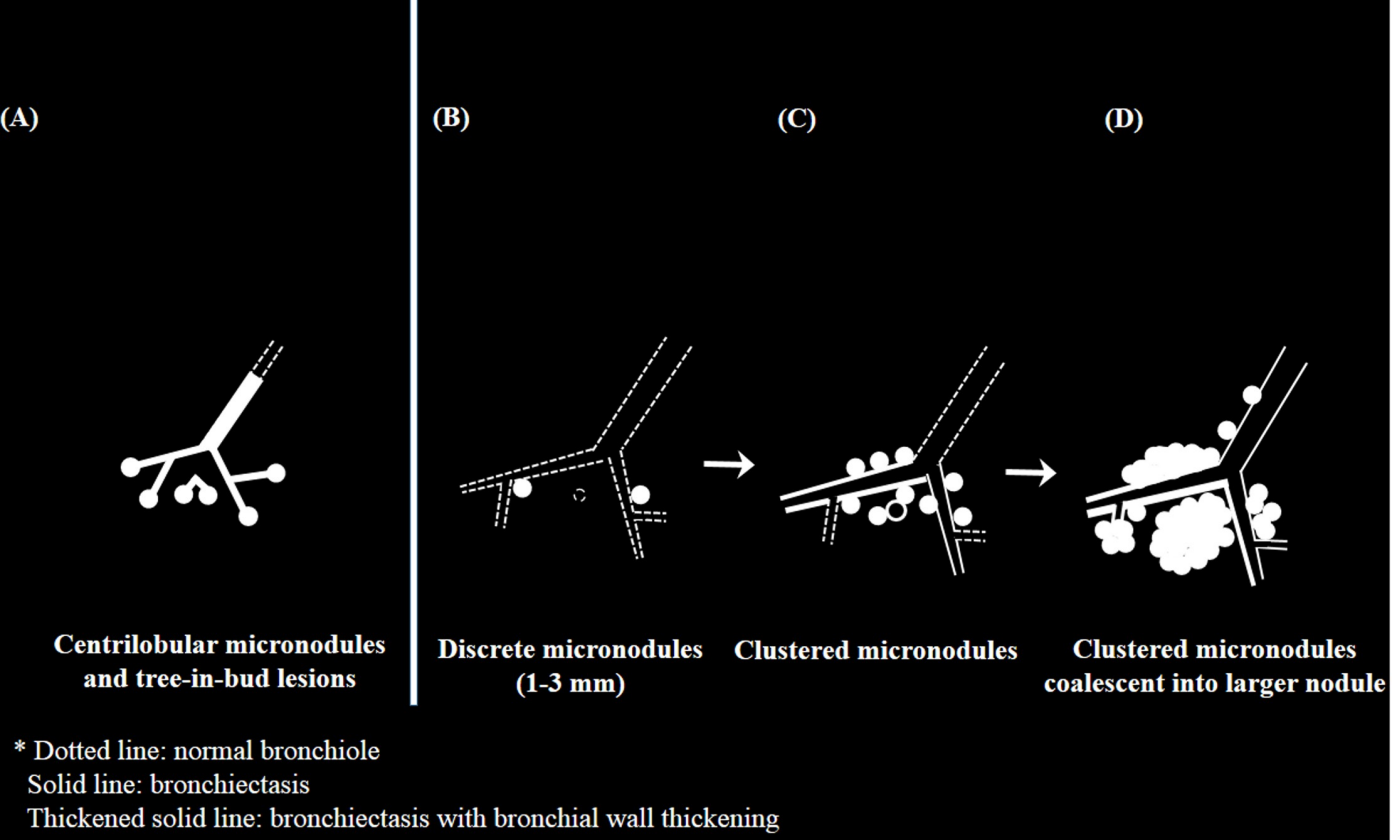
A diagram which shows characteristic findings of centrilobular micronodules and tree-in bud lesions (A) and clustered micronodules (CMs) (B, C, D). Note that CMs originated from a few discrete micronodules (1–3 mm) which primarily distributed around small airways distal to the level of segmental bronchus (B). Over time, localized aggregation of multiple discrete micronodules associated with small airway wall thickening and bronchiolectasis gradually progressed as CMs (C). Unlike tree-in-bud lesions which are correspond to small airways filled with caseous materials (A), the CMs may have association with thickened walls of small airways maintaining luminal patency. The CMs may coalescent into a larger nodule or consolidation either initially or later as CMs progress and can obscure small airways (D).

Two radiologists (J.H.H. and S.H.Y., with 5 and 12 years of chest CT interpretation, respectively) reviewed the diagnostic CT scans and reached a consensus regarding whether CMs were the most predominant CT abnormality on the diagnostic CT scan (discrepancy rates were 3.1% [26/833], and the discrepant cases were resolved through discussion). CMs were defined as a localized aggregation of multiple discrete dense micronodules (1–3 mm) spaced apart of a few millimeters or less, which primarily distributed around small airways distal to the level of the segmental bronchus. Small airways surrounded by CMs maintained luminal patency and were often dilated with thickened walls. The CMs might centrally coalesce into a larger nodule either initially or later as CMs progressed. When the CMs had a coalescence, small airways could be obscured by the conglomeration (Fig 1) [3, 7].

A tree-in-bud lesion typically consisted of small centrilobular nodules with branching opacities, which are connected to multiple branching linear structures of similar caliber originating from a single stalk, and those linear structures correspond to small airways which are filled by caseous materials [3]. Meanwhile, CMs typically lacked the similar-caliber branching connection between the nodules. The CMs might have connections between micronodules via thickened walls of small airways having luminal patency. The readers were blinded to any clinical information except for the diagnosis of active pulmonary TB.

In cases with a CM-predominant CT pattern, the two readers independently evaluated the CT findings in detail by using a segment-based scoring system, which encompassed typical (centrilobular micronodules, nodules, tree-in-bud lesions, cavity, endobronchial lesion, small airway wall thickening, bronchiolectasis/bronchiectasis) and atypical CT findings (interlobular septal thickening, ground glass opacities, consolidation, pleural effusion, random micronodules, CMs, reversed halo sign) in adults (Table 1) [8]. Centrilobular micronodules were separately assessed from tree-in-bud lesions when micronodules had typical centrilobular distribution in a secondary pulmonary lobule without branching opacity. Each item was visually categorized into four stages according to the extent, number, or presence of the findings per segment. The number of involved segments and the summation of each segmental score were analyzed to evaluate the overall disease burden per patient. Any discrepancies were resolved through consensus.

**Table 1:** A segment-based scoring system that encompassed the typical and atypical CT findings of pulmonary TB.

		Score			
	Value	0	1	2	3
**1) Typical CT findings**					
Centrilobular micronodules (< 3 mm)	Extent*	Absent	Less than 25%	25%-50%	More than 50%
Nodules (3–10 mm)	Number	Absent	1 to 3	4 to 5	More than 5
Nodules (> 1 cm)	Number	Absent	1 to 3	4 to 5	More than 5
Tree-in-bud lesions	Extent*	Absent	Less than 25%	25%-50%	More than 50%
Cavity (size)	Size	Absent	Smaller than 3 cm	3–5 cm	Larger than 5 cm
Cavity (wall)	Thickness	Absent	Thinner than 1 mm	1–5 mm	Thicker than 5 mm
Cavity	Number	Absent	1	2	More than 3
Endobronchial lesion	Presence	Absent	-	-	Present
Small airway wall thickening (lumen < 80% of total)	Number	Absent	1	2	More than 3
Bronchiolectasis/Bronchiectasis	Extent*	Absent	Less than 25%	25%-50%	More than 50%
**2) Atypical CT findings**					
Interlobular septal thickening	Extent*	Absent	Less than 25%	25%-50%	More than 50%
Ground glass opacities	Extent*	Absent	Less than 25%	25%-50%	More than 50%
Consolidation	Size	Absent	Smaller than 3 cm	3–5 cm	Larger than 5cm
Pleural effusion	Presence	Absent	Present	-	-
Random micronodules (< 3 mm)	Presence	Absent	Present	-	-
CMs	Extent*	Absent	Less than 25%	25%-50%	More than 50%
Reversed halo sign	Presence	Absent	Present	-	-

TB = Tuberculosis; CMs = clustered micronodules.

*Percentage of segment occupied by the lesion

For the patients with predominant CMs, we checked whether an incidental pre-diagnostic CT scan was available at least 3 months apart from the diagnostic CT scan. If there were multiple pre-diagnostic CT scans that could be evaluated, the oldest CT scan was chosen for the evaluation. The same readers reviewed the pre-diagnostic CT scan, using the same scoring system. Progression was defined as an increase of the segment-based score by 1 point or higher between the pre-diagnostic and diagnostic scans. The median time interval for minimal radiologic progression was defined as the difference in the segment-based score between the CT scans divided by the time interval between the CT scans. We measured the longest length of the lung parenchymal area which was involved by CMs on axial CT images.

### Pathologic evaluation

One expert pathologist (J.Y.K.) reviewed the hematoxylin and eosin-stained specimens of transthoracic or open lung biopsies that were carried out in four patients with CM-predominant CT findings whose acid-fast bacilli (AFB) smear test and PCR assay were negative for TB or lack of suspicion for TB.

### Statistical analysis

We used the Chi-square test for categorical variables and the Student *t*-test for continuous variables in comparisons between patients with predominant CMs and those without. As a cavity is a well-known predictor for sputum smear positivity, we performed a subgroup analysis sorely including patients without cavity to identify the association between patients with CMs and smear positivity, while excluding the cavity. To compare CT findings between the pre-diagnostic and diagnostic CT scans, we performed the McNemar test for categorical variables and the Wilcoxon-signed rank test for continuous variables. In all tests, *p*-values < .05 were considered to indicate statistical significance. All analyses were performed using SPSS version 22.0 (IBM Corp., Armonk, NY, USA).

## Results

### Patient characteristics

Among the 833 included patients, CMs were the most predominant diagnostic CT abnormality in 22 patients (2.6%; 95% CI, 1.8–4.0%). Among the remaining 811 patients, 763 patients did not have CMs, and CMs presented as a minor CT abnormality in 48 patients (Table 2). Of patients with predominant CMs, nine patients (40.9%) had risk factors for TB activation, and four of the nine patients had more than one risk factors (Table 2).

**Table 2:** Comparison of clinical characteristics between two groups; proven active pulmonary TB with 1) predominant CMs 2) or not.

	Active TB with predominant CMs	Active TB without predominant CMs	*p*-value
Total number of patients	22 (2.6%)	811 (97.4%)	
Age* (years)	59.8 ± 15.77	62.4 ± 17.22	.484
Sex			.053
Male	18/22 (81.8%)	499/811 (61.5%)	
Female	4/22 (18.2%)	312/811 (38.5%)	
Ever smoking	10/12 (83.3%)	290/413 (70.2%)	.304
Test for diagnosis of TB^†^			
AFB smear test positivity	1/21 (4.8%)	229/739 (31.0%)	.010
- Patients without findings of cavity	1/20 (5.0%)	137/526 (26.0%)	.034
PCR positivity	8/15 (53.3%)	513/665 (77.1%)	.940
Culture positivity	15/20 (75.0%)	548/733 (74.8%)	.981
Tissue confirm	N = 4	N = 81	
Risk factor for TB activation			
Diabetes mellitus	4/22 (18.2%)	198/811 (24.4%)	.501
Chronic renal failure	2/22 (9.0%)	51/811 (6.3%)	.645
Previous history of malignancy	6/22 (27.3%)	201/811 (24.8%)	.790
Connective tissue disease	0/22 (0.0%)	17/811 (2.0%)	1.000
Previous history of organ transplantation	1/22 (4.5%)	14/811 (1.7%)	.333
HIV infection	0/22 (0.0%)	3/811 (< 0.1%)	1.000
Total number of patients with risk factors	9/22 (40.9%)	373/811 (46.0%)	.637

Data in parentheses are percentage indicated otherwise. TB = tuberculosis; CMs = clustered micronodules; HIV = human immunodeficiency virus; AFB = acid-fast bacilli; PCR = polymerase chain reaction.

*Values are presented as arithmetic mean ± standard deviation.

^†^Not all patients have been tested for all the diagnostic tests mentioned.

Patients with predominant CMs had a significantly lower rate of AFB smear positivity (4.8% vs 31.0%; *p =* .010) (Table 2) and the AFB smear positivity was still significantly lower in the CM-predominant groups in a subgroup analysis which solely included subjects without cavity (*p* = .034). Positivity rates for PCR assays, culture, age, sex, smoking status, and risk factors for TB activation did not significantly differ between the two groups (Table 2).

### Diagnostic CT abnormalities in patients with predominant CMs

The median total CT score for all abnormalities was 10.0 (interquartile range [IQR], 6.5–14.8), and the CT score of CMs was equal to higher than that of other CT findings (median CT score, 3.0; median proportion of CT score, 36.1%). The median longest length of parenchymal involvement by CMs and number of the involved segments were 5.3 cm (IQR, 3.0–6.2 cm) and two segments, respectively. The apicoposterior segment of the left upper lobe was the most commonly involved segment (10/22, 45.4%).

The two most prevalent CT findings besides CMs were bronchiolectasis/bronchiectasis (22/22, 100%) and small airway wall thickening (21/22, 95.5%). The typical CT features of active TB were relatively infrequent: centrilobular micronodules (6/22, 27.3%), tree-in-bud lesions (7/22, 31.8%) and cavities (1/22, 4.5%) (Table 3).

**Table 3:** Radiologic features of 22 patients with predominant CMs on the diagnostic CT scan.

ID		Total CT score	CMs		Centrilobular micronodules		Nodule		Consolidation		Small airway wall thickening		Bronchiolectasis/ Bronchiectasis		Tree-in-bud lesions	
	Age (yr)						(3–10 mm)									
			CT	Seg	CT score	Seg	CT score	Seg	CT score	Seg	CT score	Seg	CT score	Seg	CT score	Seg
			score*													
1	78	10	4 (40.0)	3			1	1	1	1	2	1	2	1		
2	68	8	2 (25.0)	1			1	1	1	1	2	1	1	1		
3	88	71	25 (35.2)	17			4	4	2	1	11	8	9	9	6	2
4	79	14	6 (42.9)	5			1	1	1	1	3	2	2	2		
5	56	7	3 (42.9)	2			1	1			1	1	1	1	1	1
6	84	68	25 (36.8)	9			4	3	2	2	14	9	14	9		
7	71	12	4 (33.3)	2	1	1	1	1	1	1	2	1	1	1	2	1
8	55	10	3 (30.0)	2							2	2	2	2	2	2
9	65	5	2 (40.0)	1					1	1	1	1	1	1		
10	57	3	1 (33.3)	1							1	1	1	1		
11	45	14	5 (35.7)	3			1	1	1	1	3	3	4	4		
12	66	4	2 (50.0)	1									1	1	1	1
13	50	4	1 (25.0)	1			1	1			1	1	1	1		
14	31	12	4 (33.3)	3	1	1	2	2	1	1	2	2	2	2		
15	49	7	3 (42.9)	1			1	1			1	1	2	1		
16	54	4	1 (25.0)	1	1	1					1	1	1	1		
17	82	17	6 (35.3)	3	2	2	1	1			2	2	2	2		
18	34	8	3 (37.5)	2			1	1	1	1	1	1	1	1		
19	55	12	3 (25.0)	2			2	2	2	1	3	3	1	1	1	1
20	47	7	3 (42.9)	1	1	1	1	1			1	1	1	1		
21	58	18	8 (44.4)	4					3	3	1	1	1	1	4	4
22	44	22	8 (36.4)	4	1	1	4	3	1	1	4	3	3	3		
Total^†^			22/22 (100.0)		6/22 (27.3)		16/22 (72.7)		13/22 (59.1)		21/22 (95.5)		22/22 (100.0)		7/22 (31.8)	

None of the patients had CT features of random micronodules, interlobular septal thickening, endobronchial lesion and reversed halo sign. Eight, two, two and one patients had CT features of nodule >1 cm in size, ground glass opacities, pleural effusion and cavity, respectively. CMs = clustered micronodules; yr = years; Seg = number of involved segments.

*Data in parentheses are percentage and denote CT score of CM/total CT score.

^†^Data in parentheses are percentage and denote the number of patients having following CT findings divided by total number of patients (n = 22).

**Fig 2:**
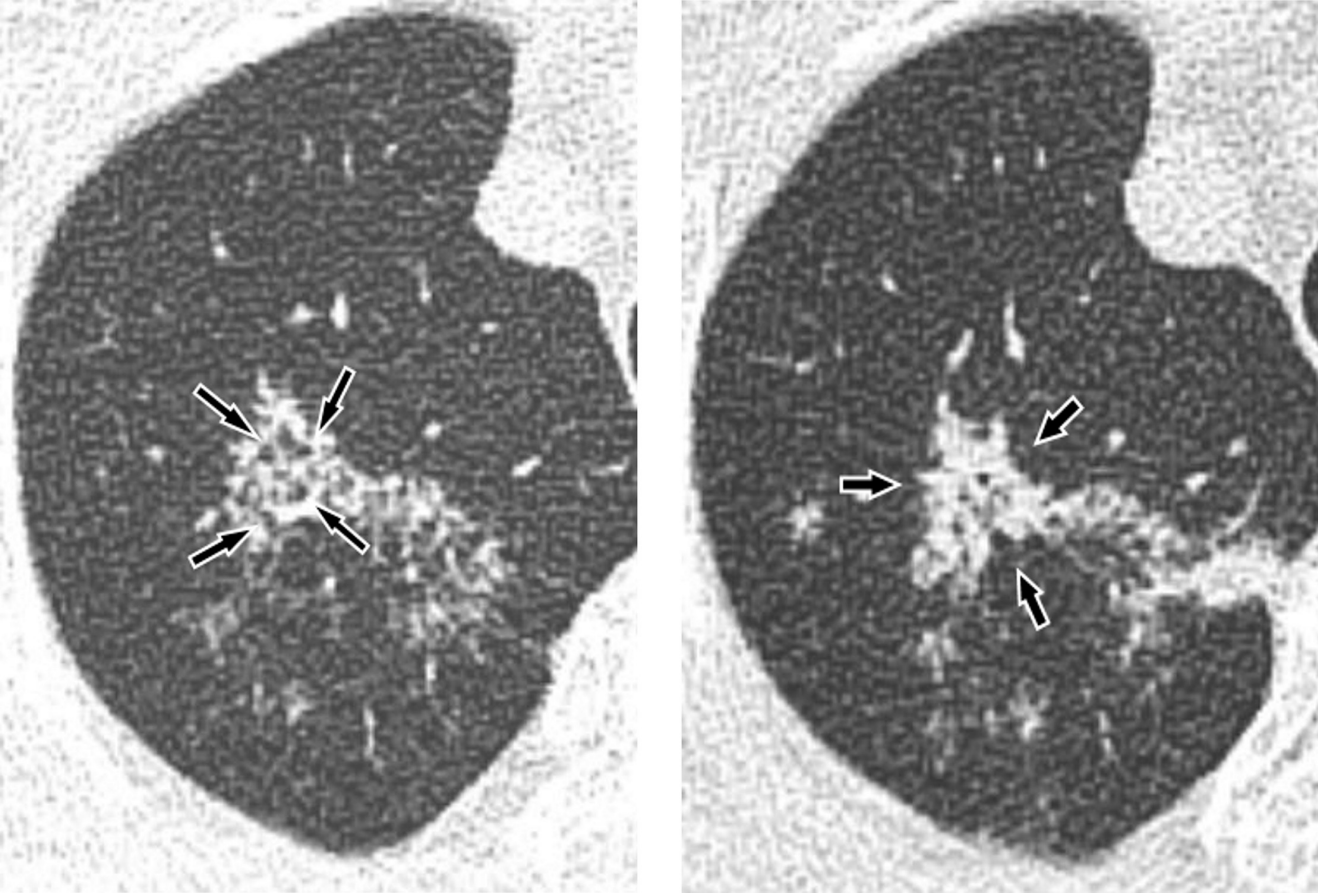
Active pulmonary tuberculosis with predominant radiologic findings of Clustered Micronodules (CMs) with large nodular opacity consist of coalescent micronodules in a 79-year-old male patient. (**A**) A pre-diagnostic CT image taken 2 months before the diagnosis of active pulmonary TB. There are CMs along the thickened dilated small airways (*arrow*) in the right upper lobe. A central coalescence is not evident at the pre-diagnostic CT scan. (**B**) A diagnostic CT image shows the progression of CMs into a large pulmonary nodular opacity with central coalescence (*arrow*), which can mimic ‘sarcoid galaxy sign’. Some of dilated small airways are obscured by the central coalescence.

Among the 22 patients, five patients had a central coalescence, mimicking a classic ‘sarcoid galaxy sign’ (Fig 2). In patients with the central coalescence, the total CT score and CT score of CMs were higher than those in patients without the central coalescences (median total CT score, 18.0 vs 8.0; median CT score of CMs, 8.0 vs 3.0, respectively). Three patients had mediastinal lymphadenopathy.

### Pre-diagnostic CT abnormalities in patients with predominant CMs

Among the 22 patients with predominant CMs, an incidental pre-diagnostic CT scan was available in 11 patients. The median interval between the pre-diagnostic and diagnostic CT scans was 49.4 months (IQR, 27.5–57.3 months), and the median time interval for minimal radiologic progression was 6.4 months (IQR, 3.0–8.2 months).

**Fig 3:**
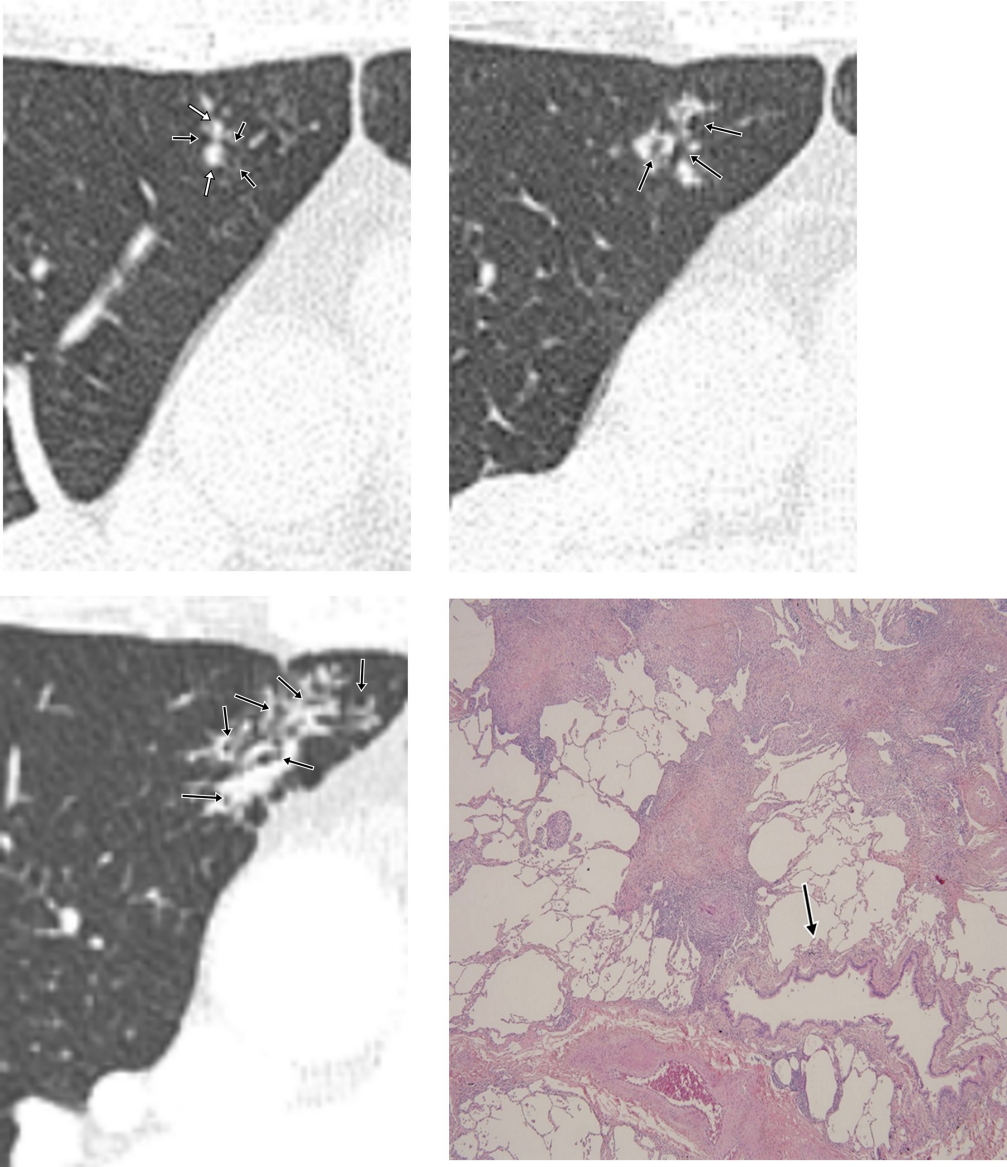
Active pulmonary tuberculosis with predominant radiologic findings of Clustered Micronodules (CMs) in a 57-year-old male patient over the course of 57 months. (**A**), A pre-diagnostic CT image taken 57 months before the diagnosis of active pulmonary TB shows a few 1–3 mm discrete micronodules (*white arrow*) which located around small airways (*black arrow*) at the anterior segment of the right upper lobe (CT score 1). (**B**), CT image obtained 42 months before the diagnosis of active pulmonary TB shows an increasing number and subsequently localized aggregation of peribronchiolar micronodules around small airway (*arrow*) in the right upper lobe, which is defined as CMs. (**C**), CT image taken at the time of diagnosis of active pulmonary TB shows progression of CMs primarily distributed along the small airways, accompanied by small airway wall thickening and dilated bronchus (*arrow*) (CT score 3). (**D**), Photograph of a histopathologic specimen (open lung biopsy) shows non-caseating granulomas confined to the peribronchiolar interstitium without evidence of airway (*arrow*) or parenchymal involvement (hematoxylin and eosin, ×40). Morphologically, the lesions are indistinguishable from those of sarcoidosis. Active pulmonary tuberculosis was confirmed by positive results of a polymerase chain reaction from resected lung tissue.

**Fig 4:**
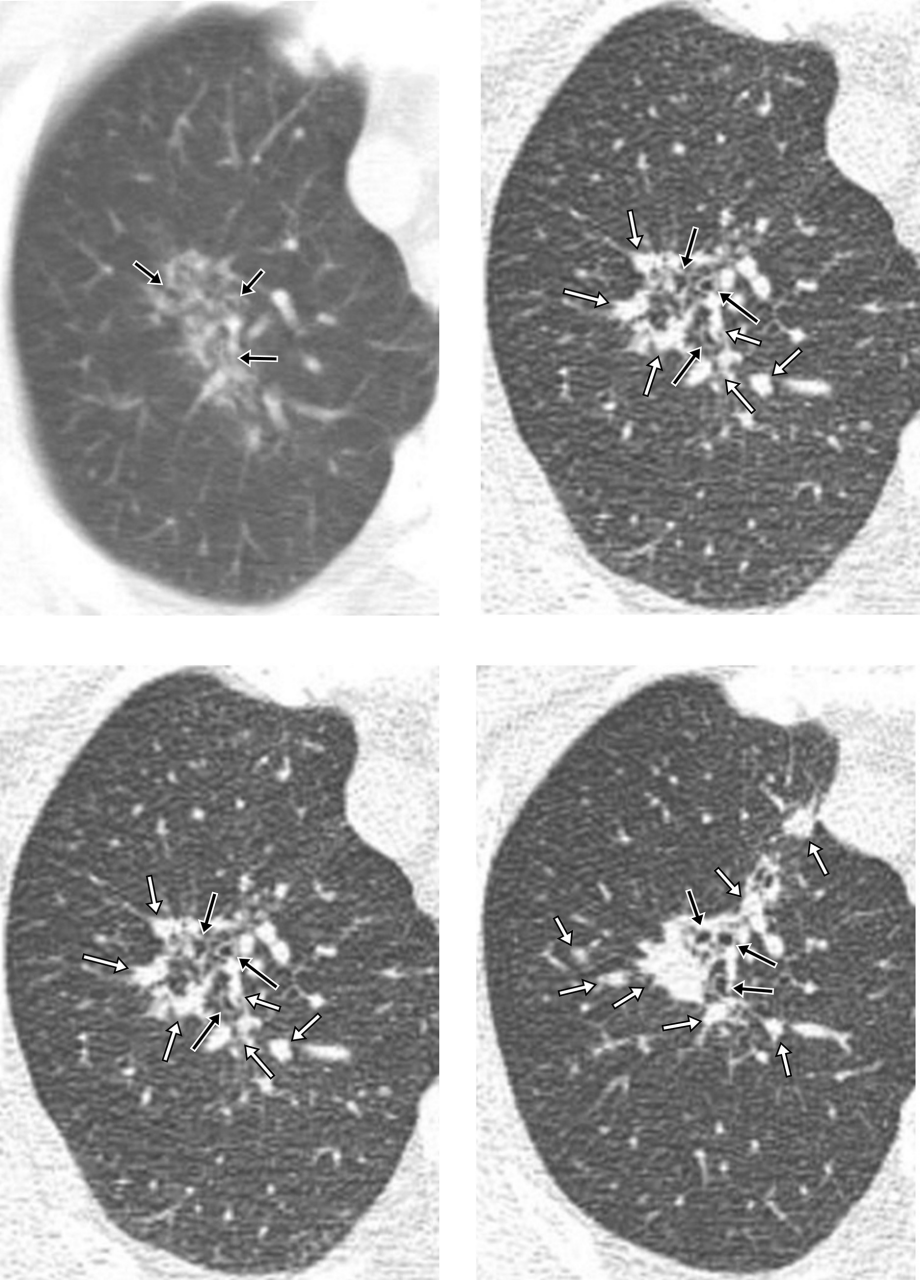
Active pulmonary tuberculosis with predominant radiologic findings of Clustered Micronodules (CMs) in a 68-year-old male patient over the course of 76 months. (**A**), A pre-diagnostic CT image taken 76 months before the diagnostic CT scan shows a focal aggregation of micronodules around the small airway with wall thickening (*arrow*) that cannot be explained by centrilobular or miliary distribution in the apical segment of the right upper lobe (CT score 3). (**B**), (**C**) **and** (**D**), Three serial CT images obtained 48 months, 13 months, and 0 month (diagnostic CT) before the diagnosis of active pulmonary TB. The CMs distributed along the bronchiolectasis (*black arrow*) were stable during follow-up until 48 months before the diagnosis, and then gradually progressed to consolidations and nodules (*white arrow*) starting 13 months before the diagnosis (CT score 8).

As the disease progressed, the median longest length of parenchymal involvement by CMs increased (pre-diagnostic CT scans, 2.9cm; diagnostic CT scans, 5.3cm). In addition, the proportion of patients with consolidation (18.2% vs 72.7%) and small airway wall thickening (45.5% vs 100.0%) significantly increased (Figs 3 and 4, Table 4). Some areas of progressed CMs were indistinguishable from bronchogenic spread of TB on the diagnostic CT scan.

**Table 4:** Comparison of radiologic characteristics between pre-diagnostic and diagnostic CT scans in patients with predominant CMs with available pre-diagnostic CT.

	Pre-diagnostic CT scan	Diagnostic CT scan	*p*-value
	N = 11	N = 11	
Median total CT score (IQR)*	3.0 (1.0–6.0)	10.0 (7.0–14.0)	.003
Median CT score (IQR) for each finding*			
Centrilobular micronodules	1.5	1.0	
Nodule (3–10 mm)	1.0 (1.0–1.0)	1.0 (1.0–3.3)	.157
Nodule (>1 cm)		1.0 (1.0–2.3)	
Consolidation	1.0	1.0 (1.0–1.8)	1.000
GGO	1.0	4.5	
Small airway wall thickening	1.0 (1.0–3.0)	2.0 (1.0–3.0)	.180
Bronchiolectasis/Bronchiectasis	1.0 (1.0–4.8)	2.0 (1.0–4.0)	.414
Tree-in-bud lesions		2.0 (1.8–3.0)	
CMs	1.0 (1.0–4.5)	4.0 (2.0–6.0)	.017
**Proportion (%) of patients with following CT findings**^†^			
Centrilobular micronodules	2/11 (18.2%)	1/11 (9.0%)	1.000
Nodule (3–10 mm)	5/11 (45.5%)	8/11 (72.7%)	.375
Nodule (>1 cm)	0/11 (0.0%)	4/11 (36.4%)	.125
Consolidation	2/11 (18.2%)	8/11 (72.7%)	.031
GGO	1/11 (9.0%)	2/11 (18.2%)	1.000
Small airway wall thickening	5/11 (45.5%)	11/11 (100.0%)	.031
Bronchiolectasis/Bronchiectasis	6/11 (54.5%)	11/11 (100.0%)	.063
Tree-in-bud lesions	0/11 (0.0%)	4/11 (36.4%)	.125
CMs	8/11 (72.7%)	11/11 (100.0%)	.250

CMs = clustered micronodules; GGO = ground glass opacities; IQR = interquartile range.

*Data are expressed as the median and interquartile range (IQR). If the number of patients with each CT finding is less than four, only the median value is presented. Two columns were marked as blank because there were no patients with those CT findings.

^†^Data are expressed as proportion and denote the number of patients having following CT findings divided by total number of patients.

Five patients with predominant CMs had a pre-diagnostic CT scan obtained more than 50 months before the diagnostic chest CT scan: CMs were the initial radiologic abnormality in three patients. In other two patients, the pre-diagnostic CT scan showed a few peribronchiolar nodules, which gradually progressed into CMs on follow-up CT scans (Fig 3).

### Pathologic findings of CMs

**Fig 5:**
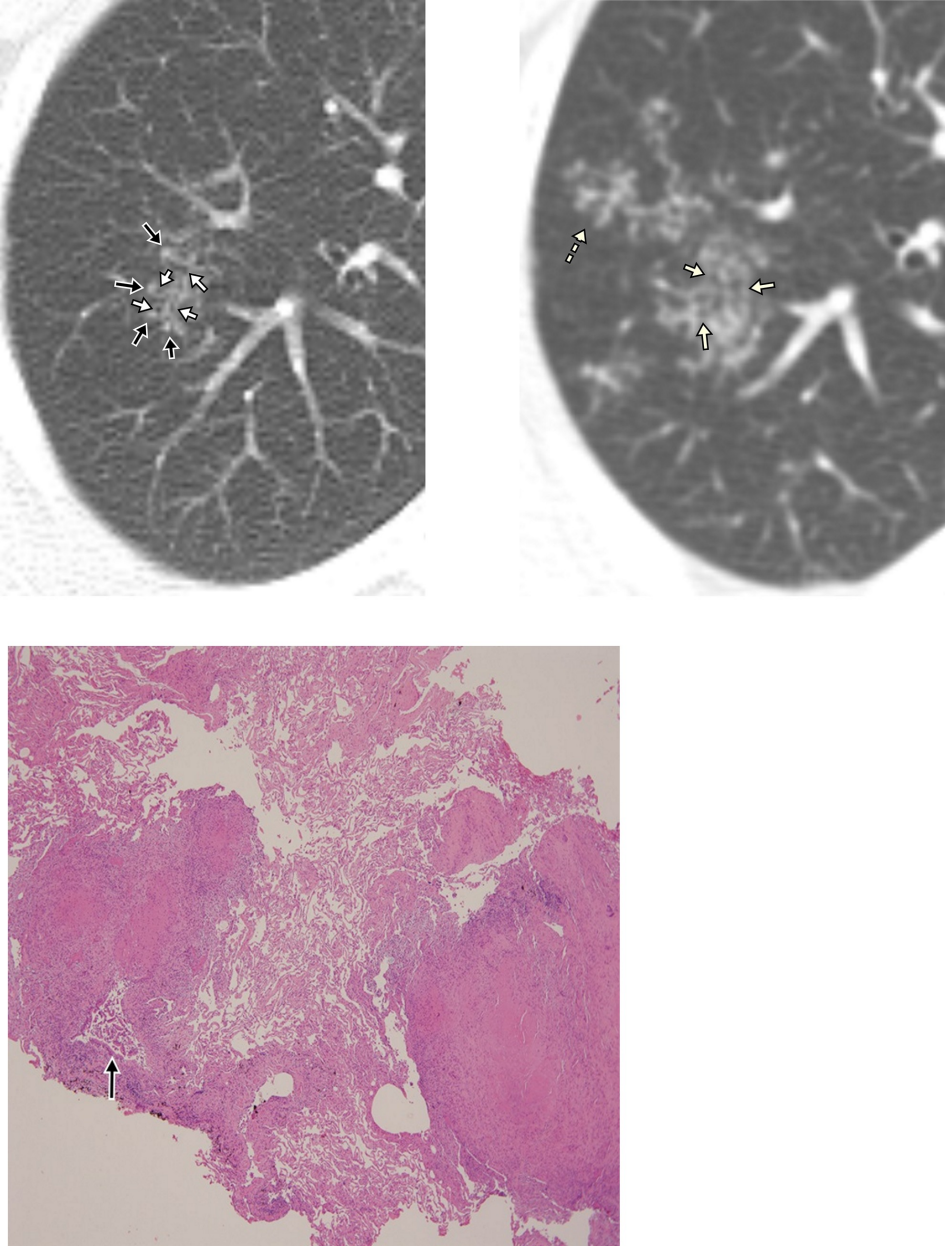
Acute pulmonary tuberculosis with predominant radiologic findings of Clustered Micronodules (CMs) in a 56-year-old female patient. (**A**), A pre-diagnostic CT image 39 months before the diagnosis of active pulmonary TB shows dense micronodules (*black arrow*) around the peribronchiolar ectatic bronchus with wall thickening (*white arrow*) in the right upper lobe (CT score 2). (**B**), Diagnostic CT images show discrete micronodules progressed into CMs, as well as interval progression of bronchiolectasis and small airway wall thickening (*white arrow*) in the right upper lobe (CT score 7). Note that tree-in bud lesions (dotted line) were developed as disease progresses, and this area was indistinguishable from bronchogenic spread of TB. (**C),** Photograph of a histopathologic specimen (open lung biopsy) shows caseating granulomas that are mainly present along peribronchial areas and invade the airway (*arrow*), with destruction of alveolar space in the lung parenchyma (hematoxylin and eosin, ×40).

Granulomas were found in all four subjects with predominant CMs who underwent transthoracic or open-lung biopsy, and the lesions were mainly located around peribronchiolar areas (Figs 3 and 5). In two patients with smaller CMs and lower CT scores (cases no. 9 and 10), granulomas had neither caseation necrosis nor invasion of the airway or alveolar space. The granulomas were confined to the peribronchiolar interstitium (Fig 3). In contrast, other two patients who had larger CMs and higher CT scores (cases no. 5 and 8) had caseating granulomas that extended into the subpleural interstitium and invaded the airway and alveolar space in a pathologic examination (Fig 5).

## Discussion

Animal experimental TB studies revealed that TB granuloma undergoes a dynamic process from non-caseating granuloma with little necrosis to caseating granuloma with the endobronchial transmission of TB following caseation necrosis [13]. Furthermore, granuloma formation on TB occurs in the interstitium rather than alveolar space at the initial stage [14]. In our study, CMs originated from few small peribronchiolar nodule which suspected as MTB granuloma [15]. The CMs indolently grew over months to years, and finally turned into a pattern of bronchogenic spread. Pathologically, smaller CMs were non-caseating granulomas confined to the peribronchiolar intersititum, whereas larger CMs were peribronchiolar caseating granulomas invading the airway and lung parenchyma. Despite the limited number of patients and scattered pathologic observations in this study, the natural history of CMs seems to follow temporal dynamics of modern understanding on TB granulomas from less-invasive non-caseating granulomas to invasive caseating granulomas [13]. The temporal evolution of TB granulomas is rarely captured in typical pulmonary TB as pulmonary TB is diagnosed mostly at the fully-brown stage with caseation necrosis, but CMs showed the evolution, as CMs were an indolent disease like a slow motion film over months to years.

CMs were present in a total of 8.4% of patients with active TB in this study; in 2.6% of patients, they were present as a major radiologic abnormality, while in 5.8%, they were present as a minor radiologic abnormality. The prevalence was lower than the rates of 9.3% to 16% reported in the relevant literature [7, 16]. The minor discordance most likely resulted from differences in the study populations and different definition of CMs regarding a central coalescence. In the former previous study [7], the study population was drawn from radiologic teaching files at a single hospital and only coalesced CMs seems to be included. The latter previous study assessed a similar study population to ours, but the percentage of patients who underwent a diagnostic CT scan was unknown [16]. In this study, 73.0% of the patients with active pulmonary TB underwent a chest CT scan at the time of diagnosis.

Traditionally, atypical radiologic manifestations of TB have been known to be prevalent in immunocompromised hosts, with a tendency to progress rapidly [5]. Although CMs can be regarded as an atypical manifestation of TB due to their rarity and preferential involvement of small airway walls with patent lumen, they had not been positively related with well-recognized risk factors for TB activation that affected the host immune status. Furthermore, considering the median time interval for minimal radiologic progression of 6.4 months, CMs seem to be a form of well-confined, but indolently progressing, pulmonary parenchymal TB disease, in contrast to typical TB evolution from a small nodule into branching opacities, lobular consolidation and cavity [17].

In patients with predominant CMs, CMs were frequently accompanied by bronchiolectasis/bronchiectasis and small airway wall thickening, indicating the bronchocentricity of the lesion. Centrilobular micronodules and tree-in-bud lesions are imaging hallmarks of the bronchogenic spread and observed in approximately 95% of active TB cases, but those lesions were less frequently observed (54.5%, 12 of 22 patients; 40.0%, 4 of 10 patients with a total CT score smaller than 10) [3, 15, 18]. In addition, cavities which are seen in 20–45% cases of active TB were rarely seen [18]. The paucity of the typical active TB findings in combination with the low rates of AFB smear positivity can indicate that the spread of TB via the airway is hampered at an early stage of TB predominantly manifesting as CMs, although CMs eventually turn into a pattern of bronchogenic spread as disease progresses.

Although pathologic findings may not represent of all 22 CM-predominant patients because of small sample size, the pathologic predilection of peribronchiolar areas is in accordance with the prevalent bronchiolectasis/bronchiectasis or small airway wall thickening on chest CT images. Interestingly, the CMs turned out to be either non-caseating or caseating granulomas and to be either non-destructing or destructing peribronchiolar lesions on pathologic evaluations. Similarly, Lee et al. [9] showed a representative case report of CMs in a patient with a minimal extent of disease and found that CMs presented as nodular aggregations of granulomas in the peribronchiolar interstitium. In contrast, when CMs were pathologically analyzed in patients with advanced TB disease, they corresponded to the aggregation of tree-in-bud lesions of impacted caseation material within the bronchioles and alveolar ducts [15]. The difference in pathologic nature of CMs between our series and previously reported cases [15] might come from the difference in the stage of TB infection. All our cases were live subjects with an early stage, while the latter cases were a post-mortem lung in advanced stage.

Although CMs in cases of active pulmonary TB can resemble the ‘sarcoid galaxy’ sign of sarcoidosis on CT images [6], mediastinal and hilar lymphadenopathy (sarcoidosis, 94% [6]; TB, 14% in this study) or ground-glass opacities surrounding CMs (sarcoidosis, 56% [6]; TB, 9% in this study) potentially help to differentiate between the two diseases. Finally, among the TB patients with CMs on their CT images, only 31% (22/70) showed CMs as a predominant manifestation, whereas 69% of cases of CMs coexisted with a predominant bronchogenic pattern of active pulmonary TB that included centrilobular micronodules and tree-in-bud lesions.

Artificial intelligence (AI) is a promising technique in medical fields, particularly for radiology. Indeed, the AI algorithm already achieved excellent performance in the detection of active tuberculosis on chest X-ray [19]. Detection of active tuberculosis on CT is still a challenging task, and CT achieved decent performance in the detection of a pulmonary nodule [20]. Although we did not use AI algorithms to automatize and objectivize to read the CT images, the AI algorithm can potentially be used to read CT images of CM-predominant TB in the near future.

Our study has several limitations. First, the number of the study population was small, and subjects were retrospectively collected at a single center, although we included patients with predominant CMs from a large consecutive cohort with active pulmonary TB. Second, pathological analyses were performed in a limited number of patients (18.2%), and it was somewhat inevitable as pathologic specimens are not needed to be obtained by invasive procedures in bacteriologically proven TB with sputum specimens. Third, some patients did not undergo sputum AFB smear tests due to a lack of suspicion for TB. Those patients received percutaneous needle or open lung biopsy because the findings of active TB on their CT scans were indistinguishable from those of lung cancer or sarcoidosis. Fourth, although our study suggested that there was no relationship between host immune status and CT findings of predominant-CMs, the number of CM-predominant patients with immunocompromised status was small. Fifth, the scoring system had limited value in evaluating subtle radiologic changes or lesions that progressed into another category that did not share the same scoring criteria. Sixth, whether CMs were predominant CT findings or not was subjectively judged based on visual inspection, but not based on specific radiologic score. To complement this, two certificated thoracic radiologists were reviewed CT images independently, and reached a consensus. Seventh, our study population mainly consisted of the elderly (mean age, 62.5 years), probably because we enrolled study population retrospectively in a single, tertiary center. Therefore, more extensive studies, including the younger age group, would be beneficial.

In conclusion, CMs were uncommon indolent forms of active pulmonary TB, which originated from small peribronchiolar nodules and slowly progressed over months to years accompanied by bronchocentric change. A proper understanding of distinctive features of patients with predominant CMs is necessary for appropriate early diagnosis and management of these patients having the active but indolent granulomatous TB disease.

## Supporting information

S1 Table (DOCX)

S2 Table (XLSX)

S1 Fig (PPTX)
