# Supplementary material for: Clustered micronodules as predominant manifestation on CT: A sign of active but indolently evolving pulmonary tuberculosis
Source: PLoS One. 2020 Apr 17;15(4):e0231537. doi: 10.1371/journal.pone.0231537 (PMC7164656; doi:10.1371/journal.pone.0231537)
Supplement: S1 Table — (DOCX) [file pone.0231537.s001.docx]

**Supplementary Table**

1. All recorded radiologic features of 22 patients with predominant CMs on the diagnostic CT scan

| **ID** | **Age (yr)** | **Sex** | **Total CT score** | **CMs** | | **Centrilobular micronodules** | | **Nodule** | | **Nodule** | | **Consoli-dation** | | **GGO** | | **Small airway wall thickening** | | **Bronchiolectasis/**  **Bronchiectasis** | | **Tree-in-bud lesions** | | **Pleural effusion** | **Cavity** | |
| --- | --- | --- | --- | --- | --- | --- | --- | --- | --- | --- | --- | --- | --- | --- | --- | --- | --- | --- | --- | --- | --- | --- | --- | --- |
|  |  |  |  |  |  |  |  | **(3**-**10 mm)** | | **(> 1 cm)** | |  |  |  |  |  |  |  |  |  |  |  |  |  |
|  |  |  |  | CT | Seg | CT score | Seg | CT score | Seg | CT score | Seg | CT score | Seg | CT score | Seg | CT score | Seg | CT score | Seg | CT score | Seg | CT score | CT score | Seg |
|  |  |  |  | score^*^ |  |  |  |  |  |  |  |  |  |  |  |  |  |  |  |  |  |  |  |  |
| 1 | 78 | M | 10 | 4 (40.0) | 3 |  |  | 1 | 1 |  |  | 1 | 1 |  |  | 2 | 1 | 2 | 1 |  |  |  |  |  |
| 2 | 68 | M | 8 | 2 (25.0) | 1 |  |  | 1 | 1 | 1 | 1 | 1 | 1 |  |  | 2 | 1 | 1 | 1 |  |  |  |  |  |
| 3 | 88 | M | 71 | 25 (35.2) | 17 |  |  | 4 | 4 | 6 | 6 | 2 | 1 | 8 | 8 | 11 | 8 | 9 | 9 | 6 | 2 |  |  |  |
| 4 | 79 | M | 14 | 6 (42.9) | 5 |  |  | 1 | 1 | 1 | 1 | 1 | 1 |  |  | 3 | 2 | 2 | 2 |  |  |  |  |  |
| 5 | 56 | F | 7 | 3 (42.9) | 2 |  |  | 1 | 1 |  |  |  |  |  |  | 1 | 1 | 1 | 1 | 1 | 1 |  |  |  |
| 6 | 84 | M | 68 | 25 (36.8) | 9 |  |  | 4 | 3 | 1 | 1 | 2 | 2 |  |  | 14 | 9 | 14 | 9 |  |  | 1 | 7 | 1 |
| 7 | 71 | M | 12 | 4 (33.3) | 2 | 1 | 1 | 1 | 1 |  |  | 1 | 1 |  |  | 2 | 1 | 1 | 1 | 2 | 1 |  |  |  |
| 8 | 55 | M | 10 | 3 (30.0) | 2 |  |  |  |  |  |  |  |  | 1 | 1 | 2 | 2 | 2 | 2 | 2 | 2 |  |  |  |
| 9 | 65 | M | 5 | 2 (40.0) | 1 |  |  |  |  |  |  | 1 | 1 |  |  | 1 | 1 | 1 | 1 |  |  |  |  |  |
| 10 | 57 | M | 3 | 1 (33.3) | 1 |  |  |  |  |  |  |  |  |  |  | 1 | 1 | 1 | 1 |  |  |  |  |  |
| 11 | 45 | F | 14 | 5 (35.7) | 3 |  |  | 1 | 1 |  |  | 1 | 1 |  |  | 3 | 3 | 4 | 4 |  |  |  |  |  |
| 12 | 66 | M | 4 | 2 (50.0) | 1 |  |  |  |  |  |  |  |  |  |  |  |  | 1 | 1 | 1 | 1 |  |  |  |
| 13 | 50 | F | 4 | 1 (25.0) | 1 |  |  | 1 | 1 |  |  |  |  |  |  | 1 | 1 | 1 | 1 |  |  |  |  |  |
| 14 | 31 | F | 12 | 4 (33.3) | 3 | 1 | 1 | 2 | 2 |  |  | 1 | 1 |  |  | 2 | 2 | 2 | 2 |  |  |  |  |  |
| 15 | 49 | M | 7 | 3 (42.9) | 1 |  |  | 1 | 1 |  |  |  |  |  |  | 1 | 1 | 2 | 1 |  |  |  |  |  |
| 16 | 54 | M | 4 | 1 (25.0) | 1 | 1 | 1 |  |  |  |  |  |  |  |  | 1 | 1 | 1 | 1 |  |  |  |  |  |
| 17 | 82 | M | 17 | 6 (35.3) | 3 | 2 | 2 | 1 | 1 | 3 | 3 |  |  |  |  | 2 | 2 | 2 | 2 |  |  | 1 |  |  |
| 18 | 34 | M | 8 | 3 (37.5) | 2 |  |  | 1 | 1 | 1 | 1 | 1 | 1 |  |  | 1 | 1 | 1 | 1 |  |  |  |  |  |
| 19 | 55 | M | 12 | 3 (25.0) | 2 |  |  | 2 | 2 |  |  | 2 | 1 |  |  | 3 | 3 | 1 | 1 | 1 | 1 |  |  |  |
| 20 | 47 | M | 7 | 3 (42.9) | 1 | 1 | 1 | 1 | 1 |  |  |  |  |  |  | 1 | 1 | 1 | 1 |  |  |  |  |  |
| 21 | 58 | M | 18 | 8 (44.4) | 4 |  |  |  |  | 1 | 1 | 3 | 3 |  |  | 1 | 1 | 1 | 1 | 4 | 4 |  |  |  |
| 22 | 44 | M | 22 | 8 (36.4) | 4 | 1 | 1 | 4 | 3 | 1 | 1 | 1 | 1 |  |  | 4 | 3 | 3 | 3 |  |  |  |  |  |

CMs = clustered micronodules; yr = years; Seg = number of involved segments.

^*^Data in parentheses are percentage, and denote CT score of CM/total CT score.

**2) Evolution of CT findings before initiation of TB treatment in 11 patients with predominant radiologic pattern of CM**

| **ID** | **Total CT score** | | **Time to interval (m)** | **Median time interval for minimal progression (m)** | **CM** | | **Centrilobular micronodules** | | **Nodule** | | **Nodule** | | **Consolidation** | | **GGO** | | **Small airway wall thickening** | | **Bronchiolectasis/**  **Bronchiectasis** | | **Tree-in-bud lesions** | | **Interval developed findings** |
| --- | --- | --- | --- | --- | --- | --- | --- | --- | --- | --- | --- | --- | --- | --- | --- | --- | --- | --- | --- | --- | --- | --- | --- |
|  |  |  |  |  |  |  |  |  | **(3**-**10 mm)** | | **(> 1 cm)** | |  |  |  |  |  |  |  |  |  |  |  |
|  | **P** | **D** |  |  | **CT score** | | **CT score** | | **CT score** | | **CT score** | | **CT score** | | **CT score** | | **CT score** | | **CT score** | |  |  |  |
|  |  |  |  |  | **P** | **D** | **P** | **D** | **P** | **D** | **P** | **D** | **P** | **D** | **P** | **D** | **P** | **D** | **P** | **D** | **P** | **D** |  |
| 1 | 6 | 10 | 27.5 | 6.9 | 3 | 4 |  |  | 1 | 1 |  |  | 1 | 1 |  |  |  | 2 | 1 | 2 |  |  |  |
| 2 | 3 | 8 | 76.1 | 15.2 | 1 | 2 |  |  |  | 1 |  | 1 |  | 1 |  |  | 1 | 2 | 1 | 1 |  |  |  |
| 3 | 4 | 71 | 53.7 | 0.8 | 1 | 25 |  |  | 1 | 4 |  | 6 |  | 2 |  | 8 | 1 | 11 | 1 | 9 |  | 6 |  |
| 4 | 2 | 14 | 35.9 | 3 |  | 6 | 2 |  |  | 1 |  | 1 |  | 1 |  |  |  | 3 |  | 2 |  |  |  |
| 5 | 1 | 7 | 49.4 | 8.2 | 1 | 3 |  |  |  | 1 |  |  |  |  |  |  |  | 1 |  | 1 |  | 1 |  |
| 6 | 37 | 68 | 57.3 | 2.5 | 18 | 25 |  |  | 1 | 4 |  | 1 |  | 2 |  |  | 8 | 14 | 10 | 14 |  |  | cavity, pleural effusion |
| 7 | 2 | 12 | 75.4 | 7.5 |  | 4 | 1 | 1 | 1 | 1 |  |  |  | 1 |  |  |  | 2 |  | 1 |  | 2 |  |
| 8 | 1 | 10 | 48.4 | 5.4 | 1 | 3 |  |  |  |  |  |  |  |  |  | 1 |  | 2 |  | 2 |  | 2 |  |
| 9 | 4 | 5 | 5.9 | 5.9 | 1 | 2 |  |  |  |  |  |  |  | 1 | 1 |  | 1 | 1 | 1 | 1 |  |  |  |
| 10 | 1 | 3 | 57.3 | 28.7 |  | 1 |  |  | 1 |  |  |  |  |  |  |  |  | 1 |  | 1 |  |  |  |
| 11 | 12 | 14 | 12.8 | 6.4 | 5 | 5 |  |  |  | 1 |  |  | 1 | 1 |  |  | 3 | 3 | 3 | 4 |  |  |  |

m = month; P = pre-diagnostic CT scan; D = diagnostic CT scan
