# Supplementary material for: Clustered micronodules as predominant manifestation on CT: A sign of active but indolently evolving pulmonary tuberculosis
Source: PLoS One. 2020 Apr 17;15(4):e0231537. doi: 10.1371/journal.pone.0231537 (PMC7164656; doi:10.1371/journal.pone.0231537)
Supplement: S1 Fig — (PPTX) [file pone.0231537.s003.pptx]

## Slide 1
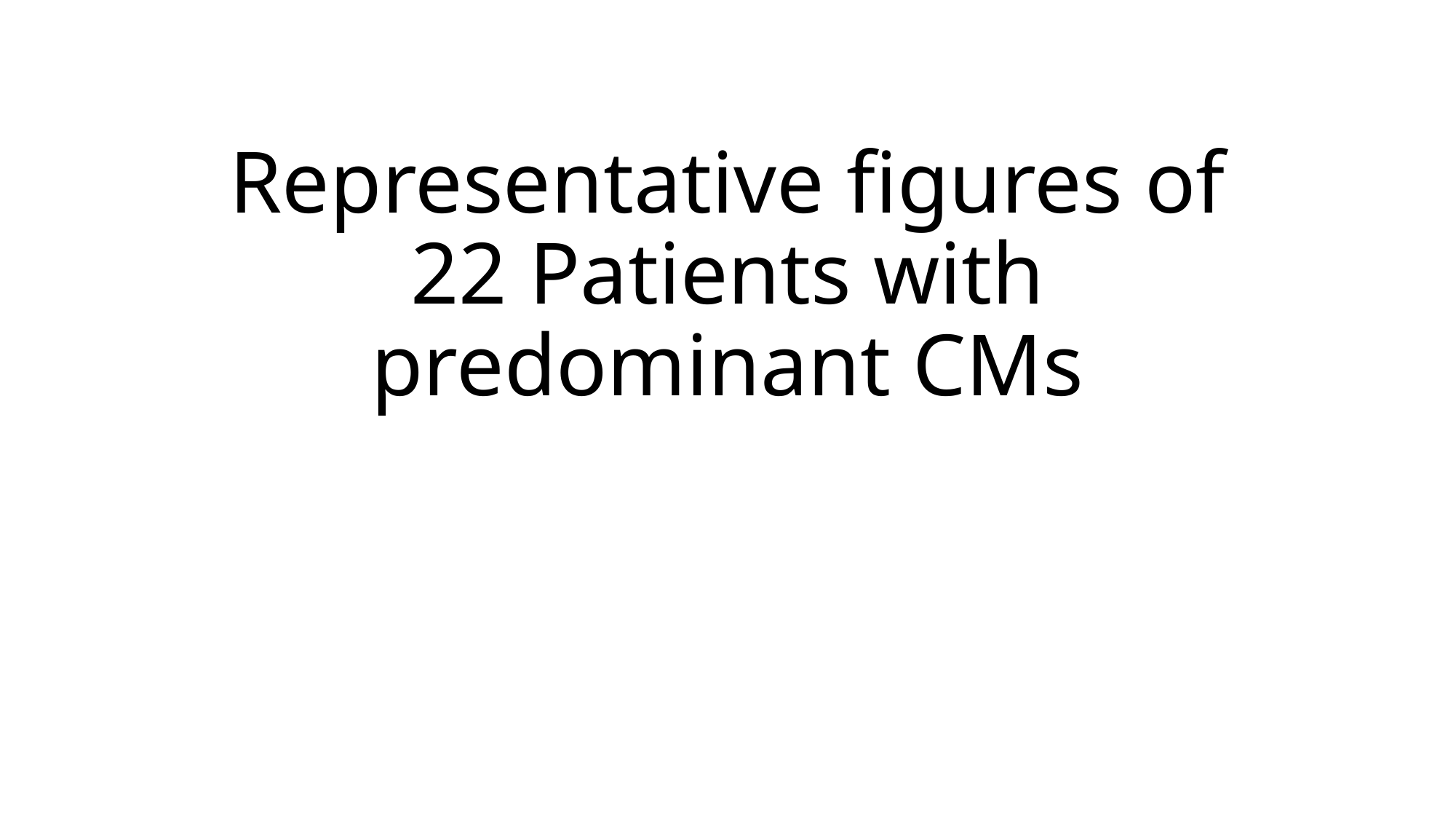

# Representative figures of 22 Patients with predominant CMs

## Slide 2
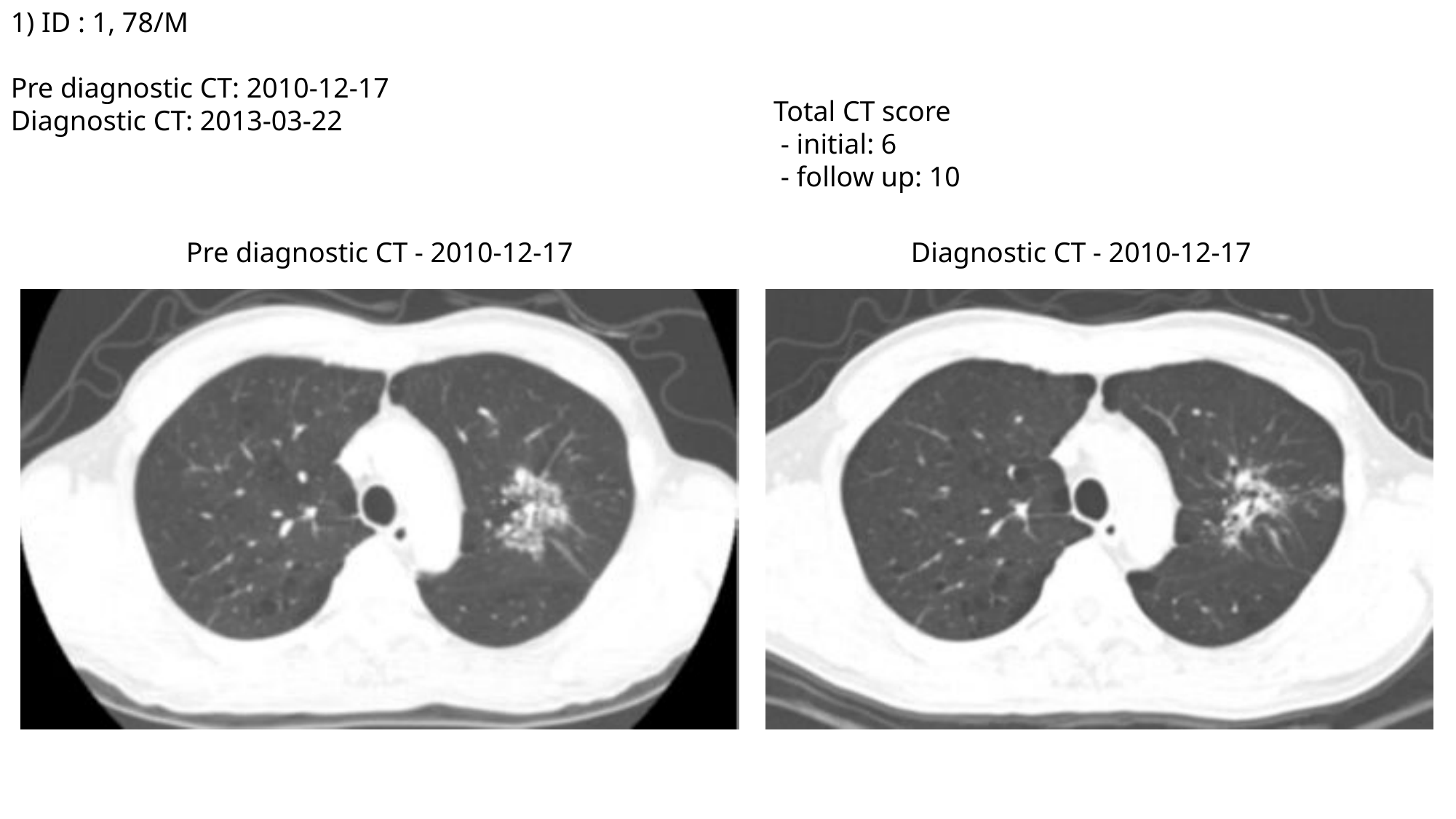

1) ID : 1, 78/M
Pre diagnostic CT: 2010-12-17
Diagnostic CT: 2013-03-22
Total CT score
 - initial: 6
 - follow up: 10
Pre diagnostic CT - 2010-12-17
Diagnostic CT - 2010-12-17

## Slide 3
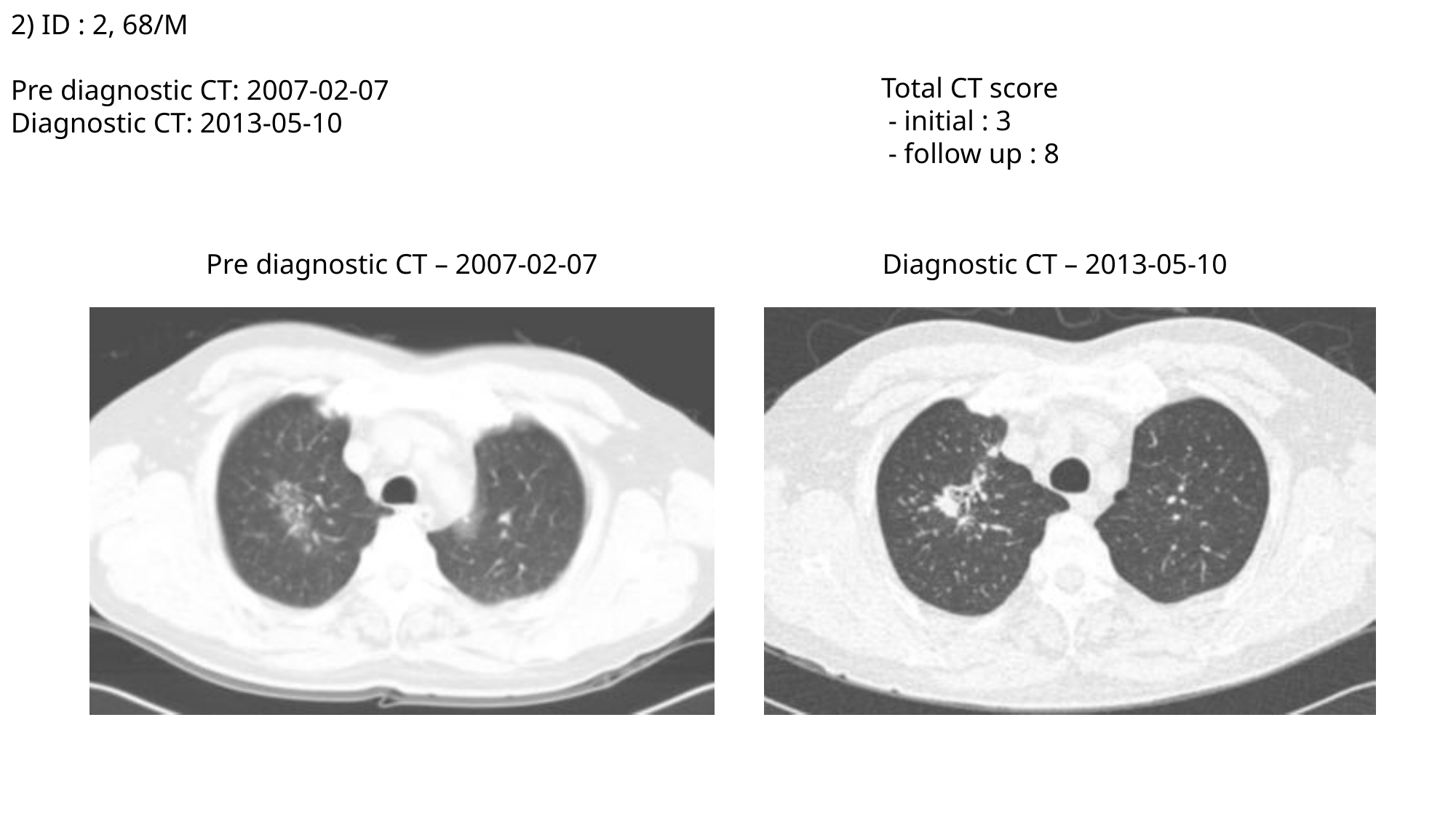

2) ID : 2, 68/M
Pre diagnostic CT: 2007-02-07
Diagnostic CT: 2013-05-10
Total CT score
 - initial : 3
 - follow up : 8
Pre diagnostic CT – 2007-02-07
Diagnostic CT – 2013-05-10

## Slide 4
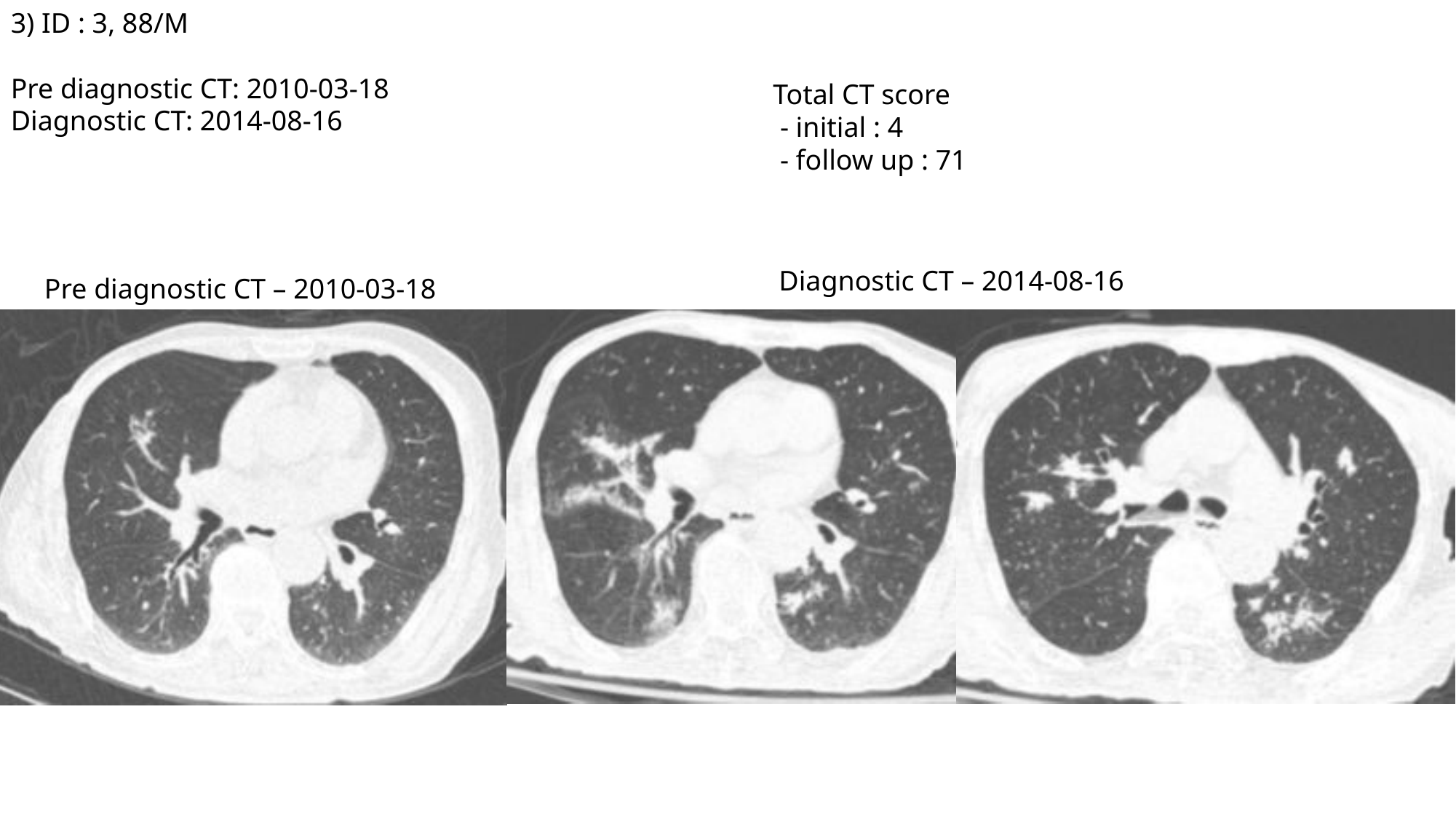

3) ID : 3, 88/M
Pre diagnostic CT: 2010-03-18
Diagnostic CT: 2014-08-16
Total CT score
 - initial : 4
 - follow up : 71
Diagnostic CT – 2014-08-16
Pre diagnostic CT – 2010-03-18

## Slide 5
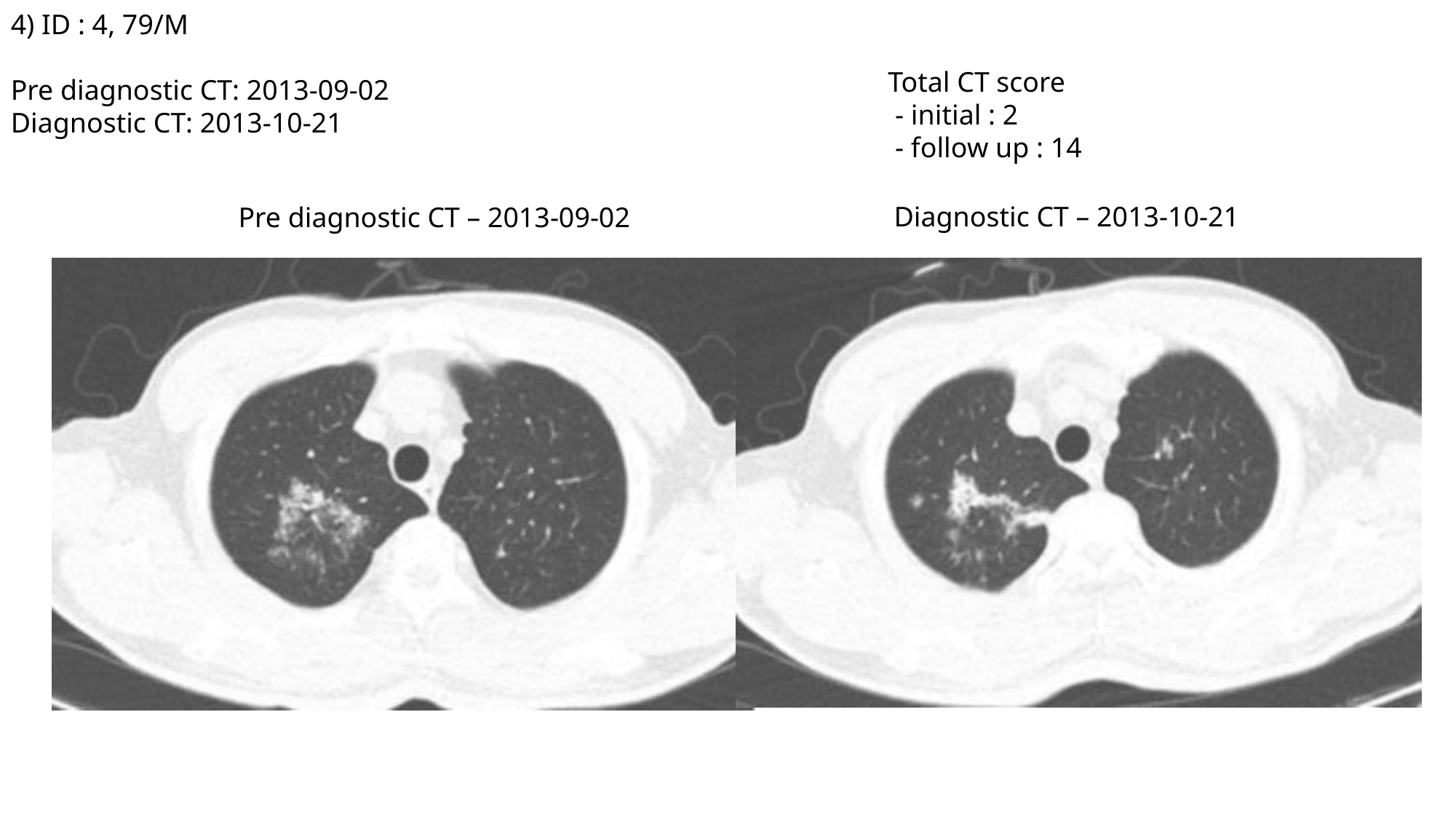

4) ID : 4, 79/M
Pre diagnostic CT: 2013-09-02
Diagnostic CT: 2013-10-21
Total CT score
 - initial : 2
 - follow up : 14
Diagnostic CT – 2013-10-21
Pre diagnostic CT – 2013-09-02

## Slide 6
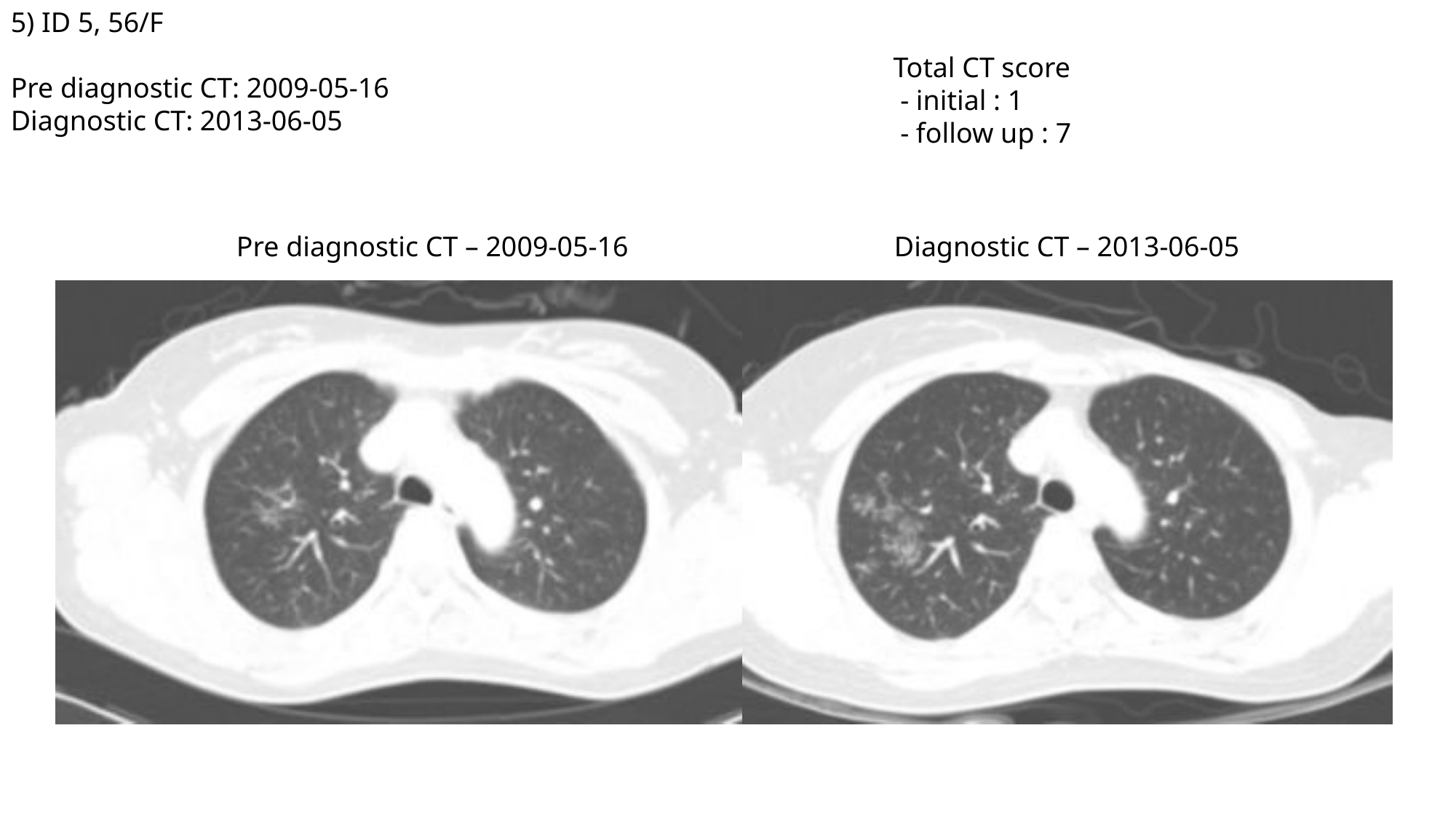

5) ID 5, 56/F
Pre diagnostic CT: 2009-05-16
Diagnostic CT: 2013-06-05
Total CT score
 - initial : 1
 - follow up : 7
Pre diagnostic CT – 2009-05-16
Diagnostic CT – 2013-06-05

## Slide 7
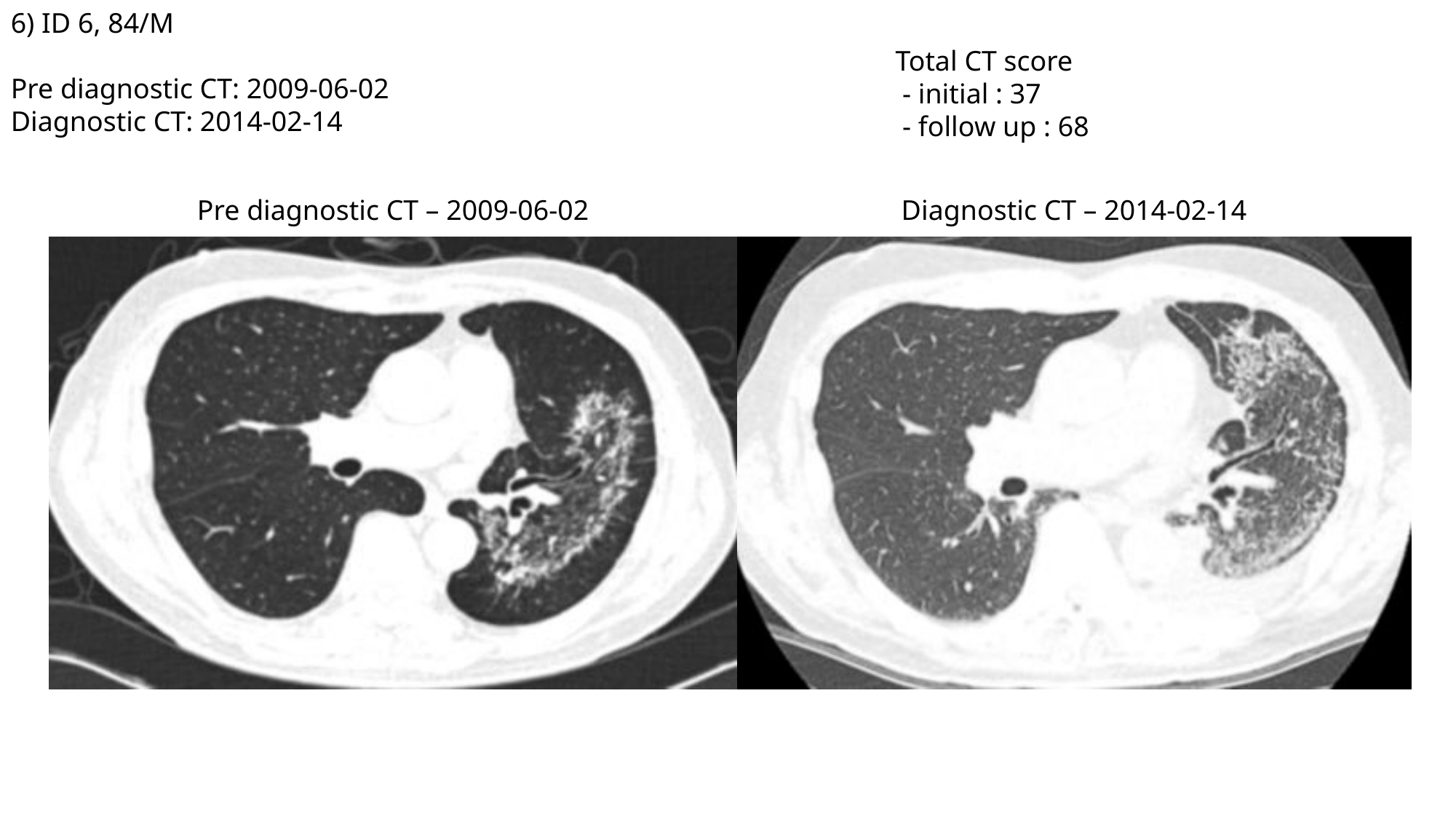

6) ID 6, 84/M
Pre diagnostic CT: 2009-06-02
Diagnostic CT: 2014-02-14
Total CT score
 - initial : 37
 - follow up : 68
Pre diagnostic CT – 2009-06-02
Diagnostic CT – 2014-02-14

## Slide 8
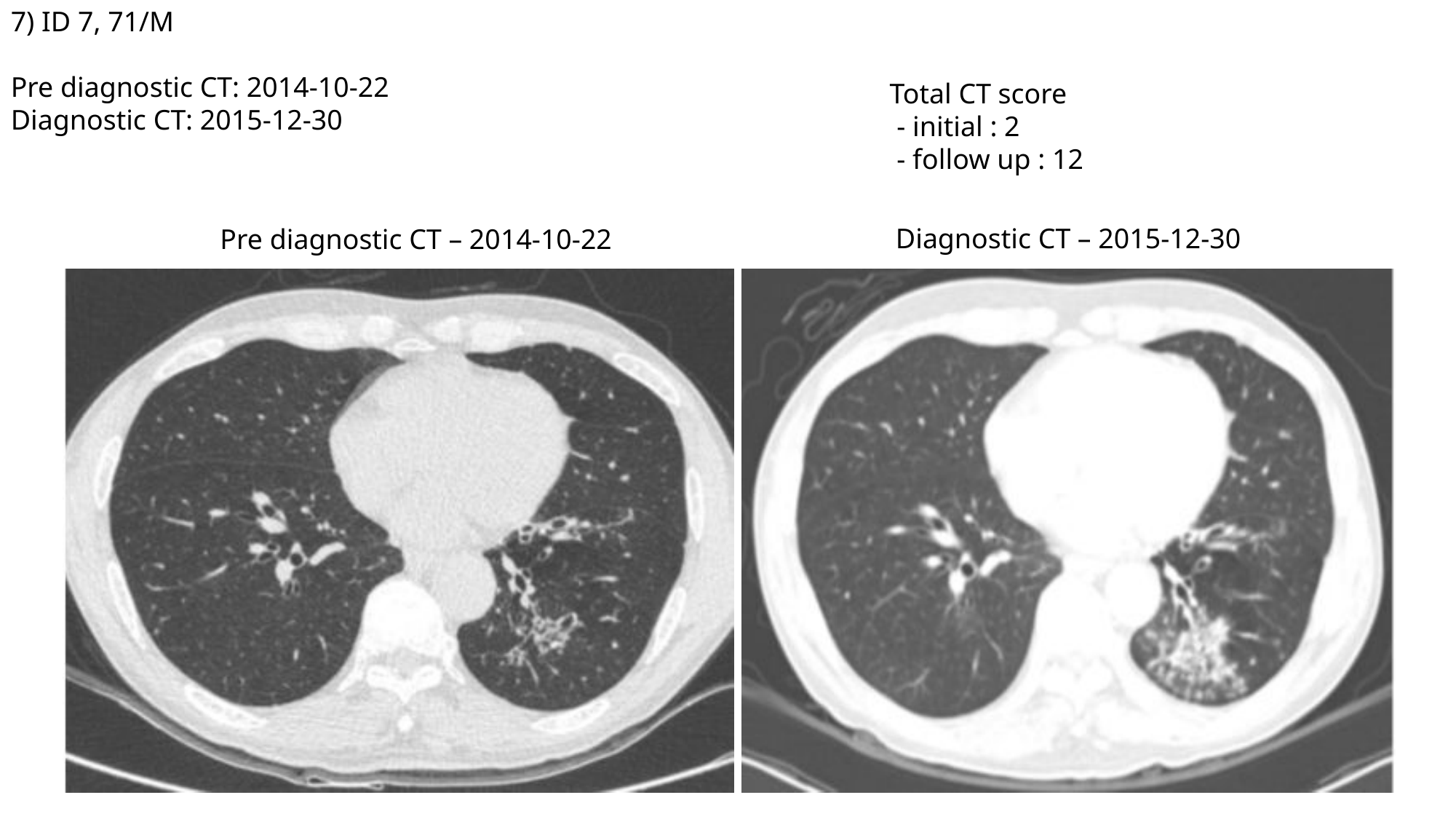

7) ID 7, 71/M
Pre diagnostic CT: 2014-10-22
Diagnostic CT: 2015-12-30
Total CT score
 - initial : 2
 - follow up : 12
Diagnostic CT – 2015-12-30
Pre diagnostic CT – 2014-10-22

## Slide 9
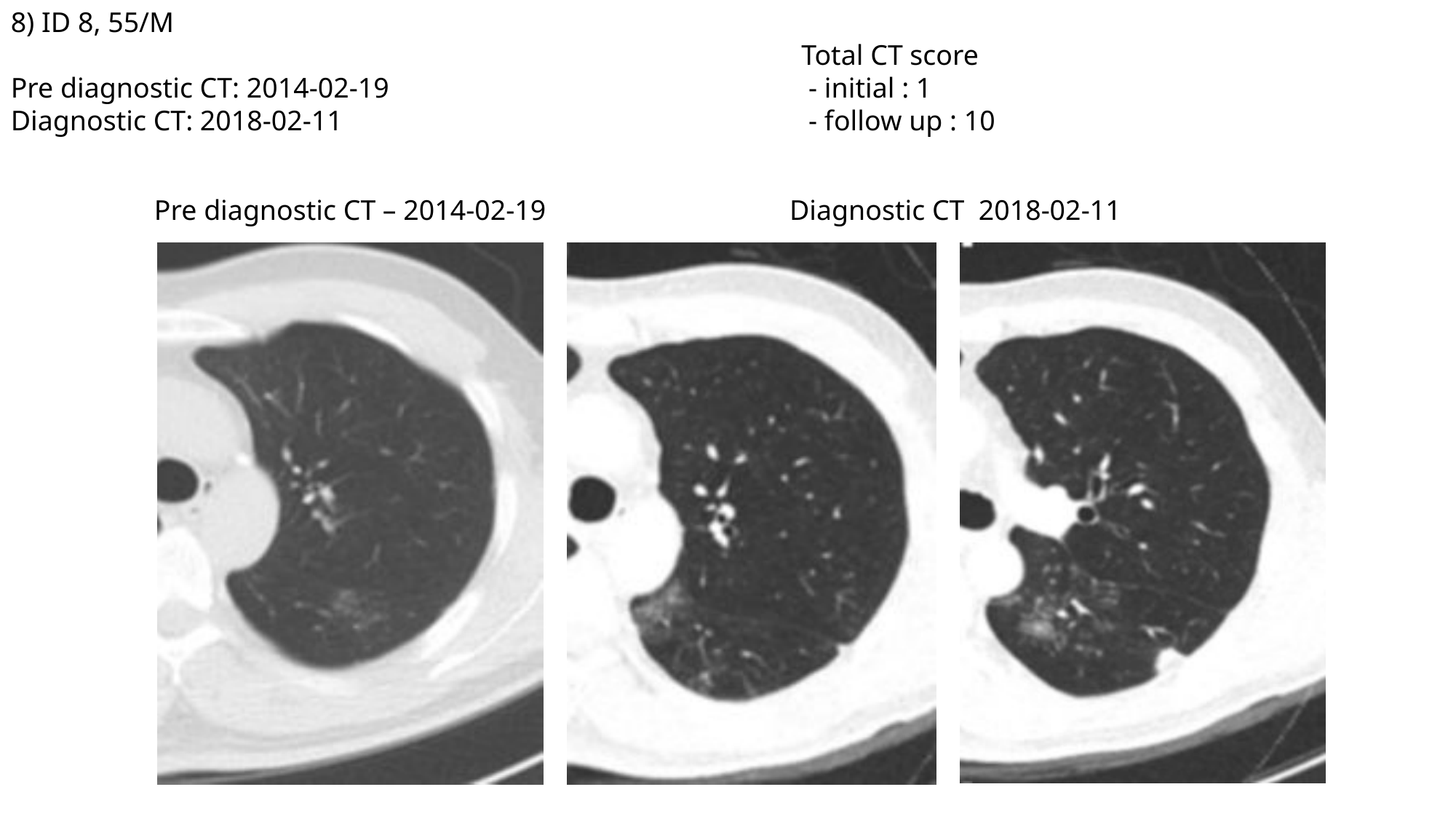

8) ID 8, 55/M
Pre diagnostic CT: 2014-02-19
Diagnostic CT: 2018-02-11
Total CT score
 - initial : 1
 - follow up : 10
Pre diagnostic CT – 2014-02-19
Diagnostic CT 2018-02-11

## Slide 10
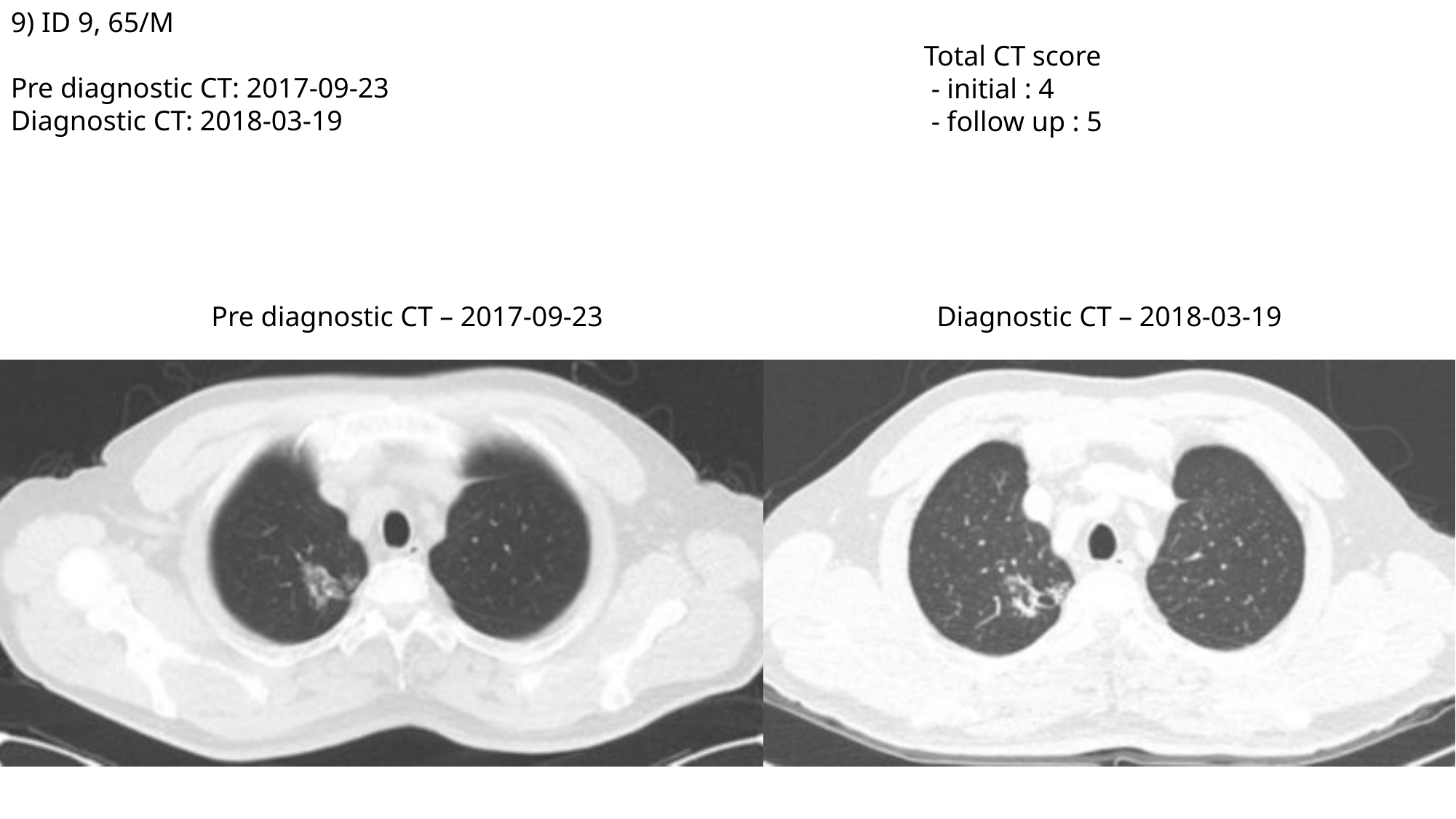

9) ID 9, 65/M
Pre diagnostic CT: 2017-09-23
Diagnostic CT: 2018-03-19
Total CT score
 - initial : 4
 - follow up : 5
Pre diagnostic CT – 2017-09-23
Diagnostic CT – 2018-03-19

## Slide 11
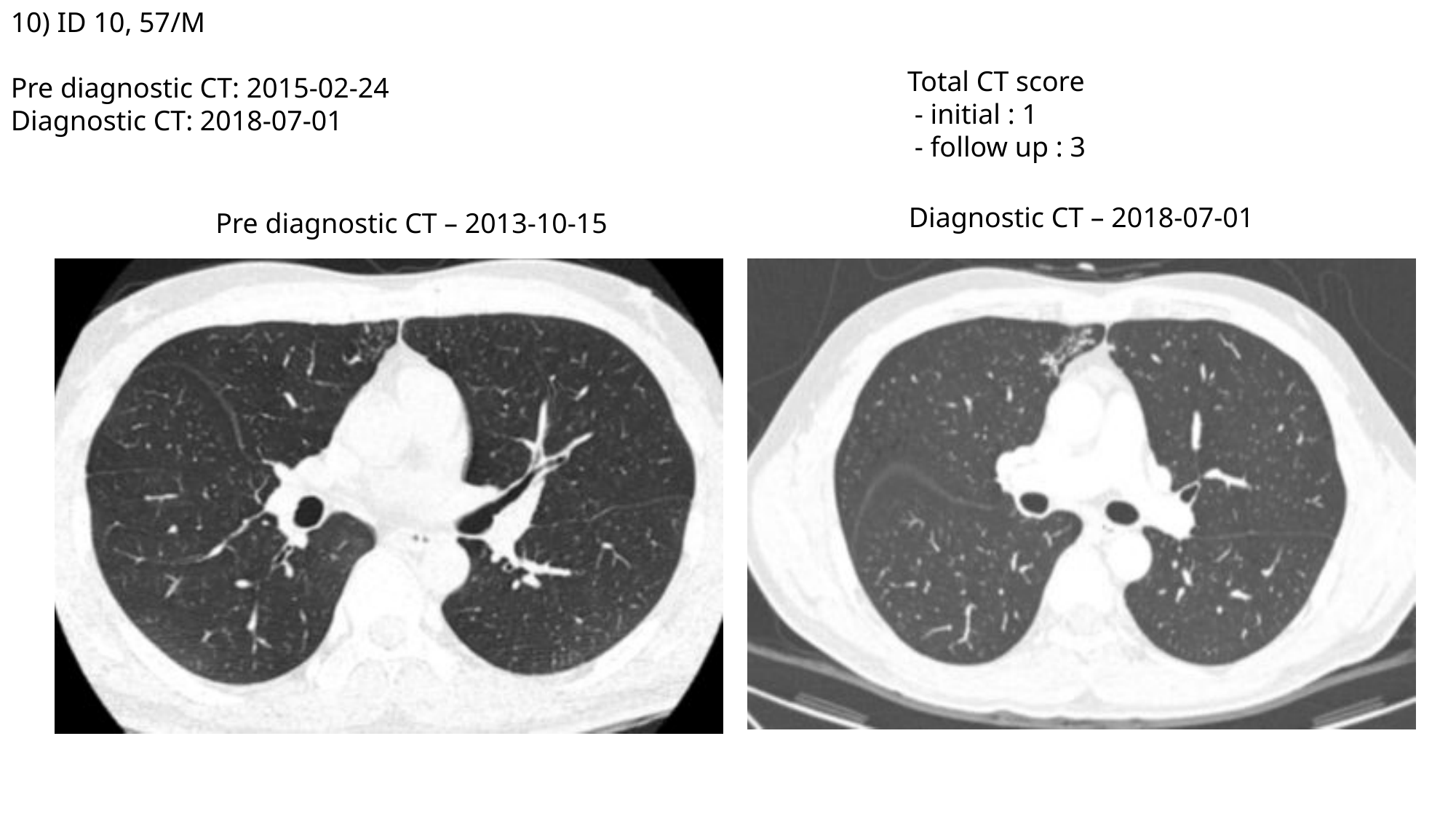

10) ID 10, 57/M
Pre diagnostic CT: 2015-02-24
Diagnostic CT: 2018-07-01
Total CT score
 - initial : 1
 - follow up : 3
Diagnostic CT – 2018-07-01
Pre diagnostic CT – 2013-10-15

## Slide 12
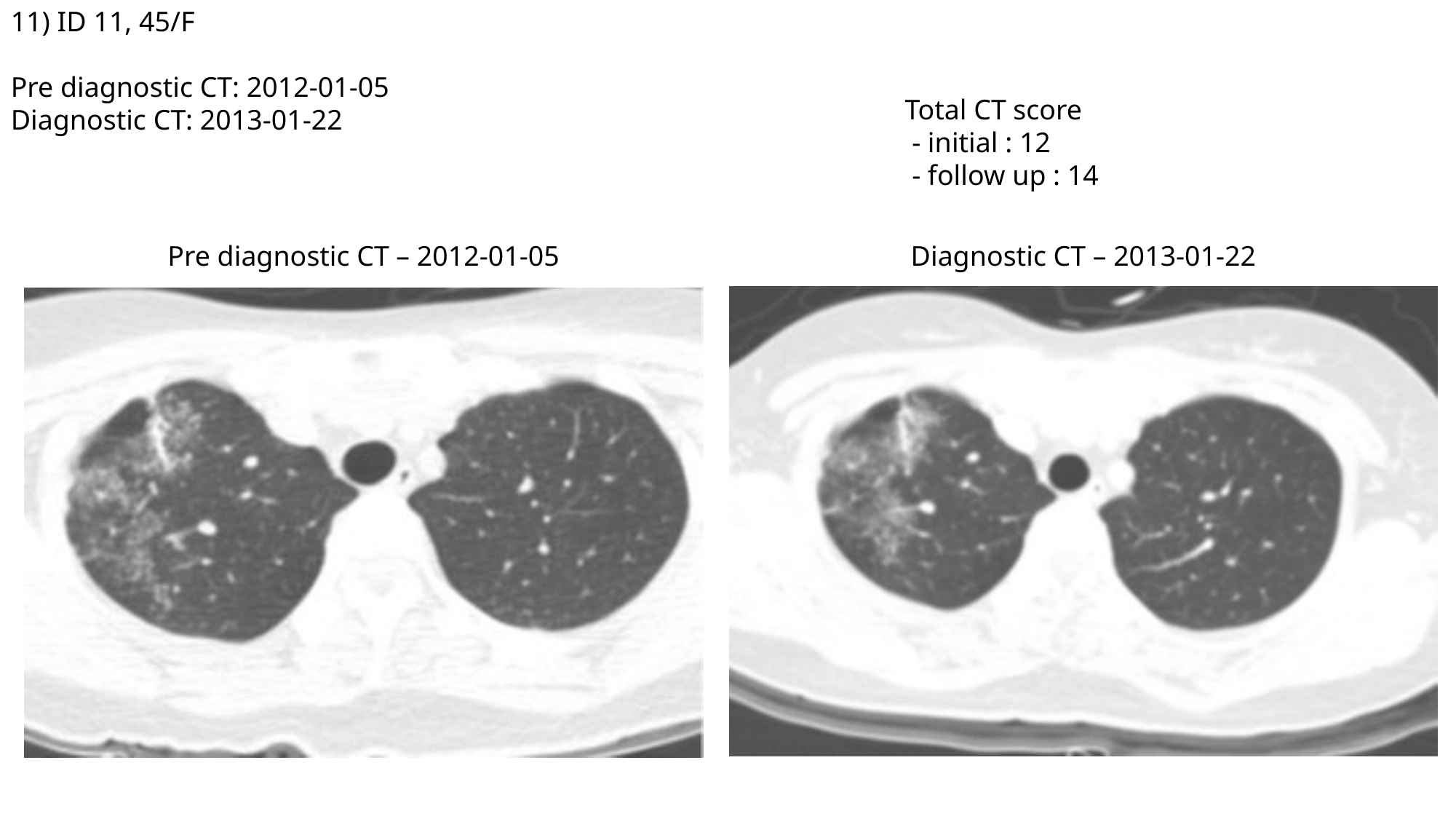

11) ID 11, 45/F
Pre diagnostic CT: 2012-01-05
Diagnostic CT: 2013-01-22
Total CT score
 - initial : 12
 - follow up : 14
Pre diagnostic CT – 2012-01-05
Diagnostic CT – 2013-01-22

## Slide 13
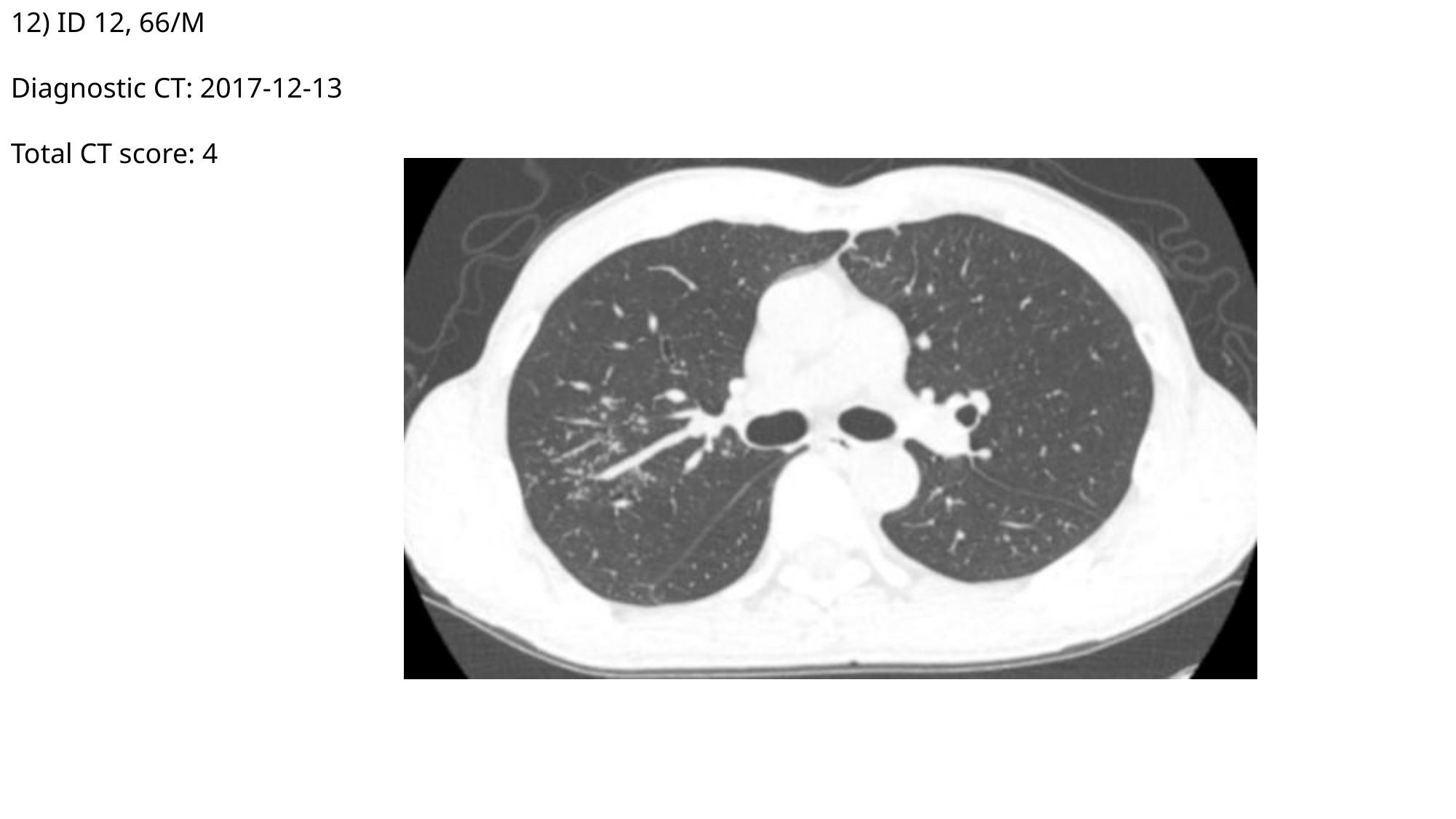

12) ID 12, 66/M
Diagnostic CT: 2017-12-13
Total CT score: 4

## Slide 14
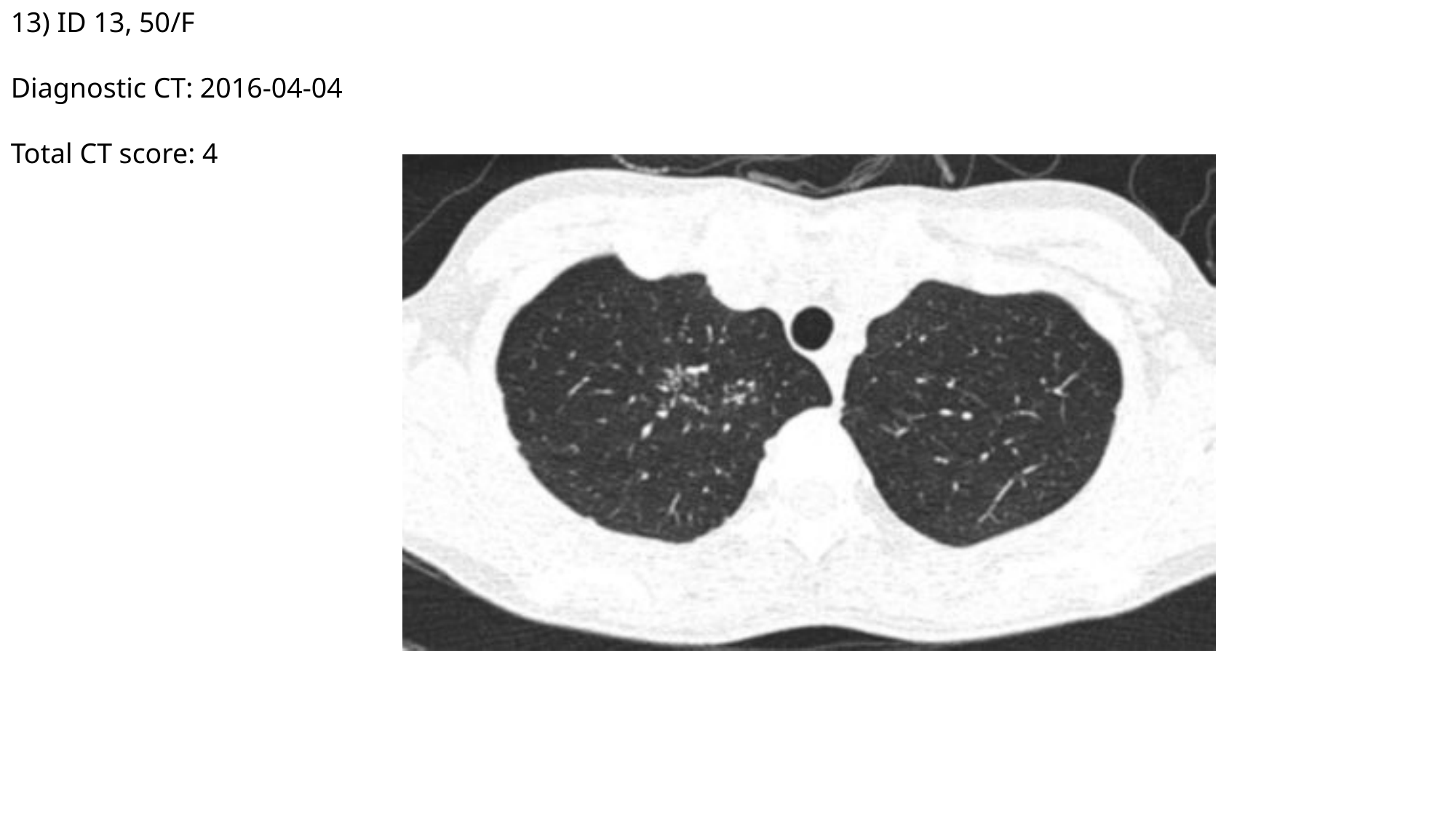

13) ID 13, 50/F
Diagnostic CT: 2016-04-04
Total CT score: 4

## Slide 15
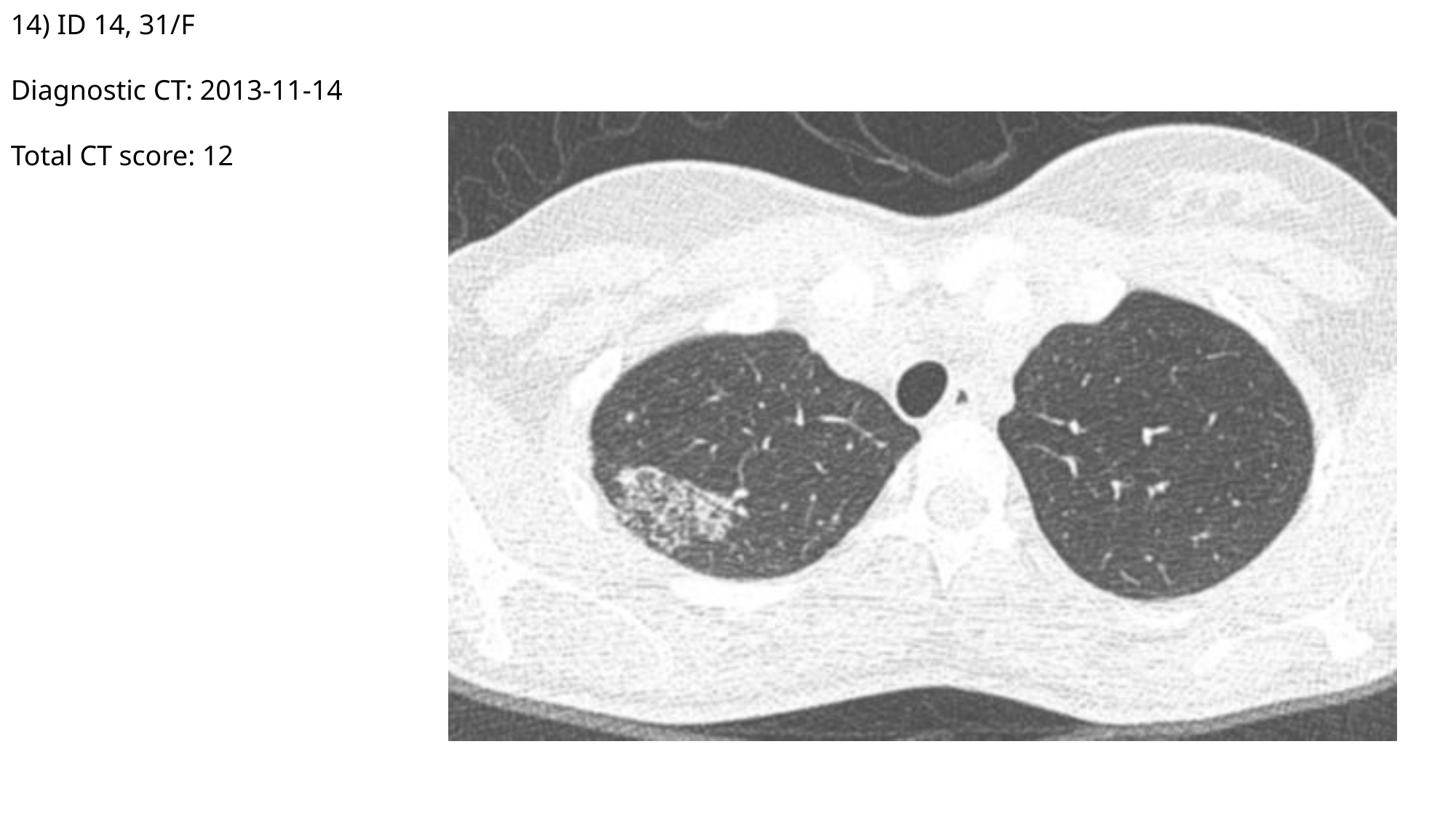

14) ID 14, 31/F
Diagnostic CT: 2013-11-14
Total CT score: 12

## Slide 16
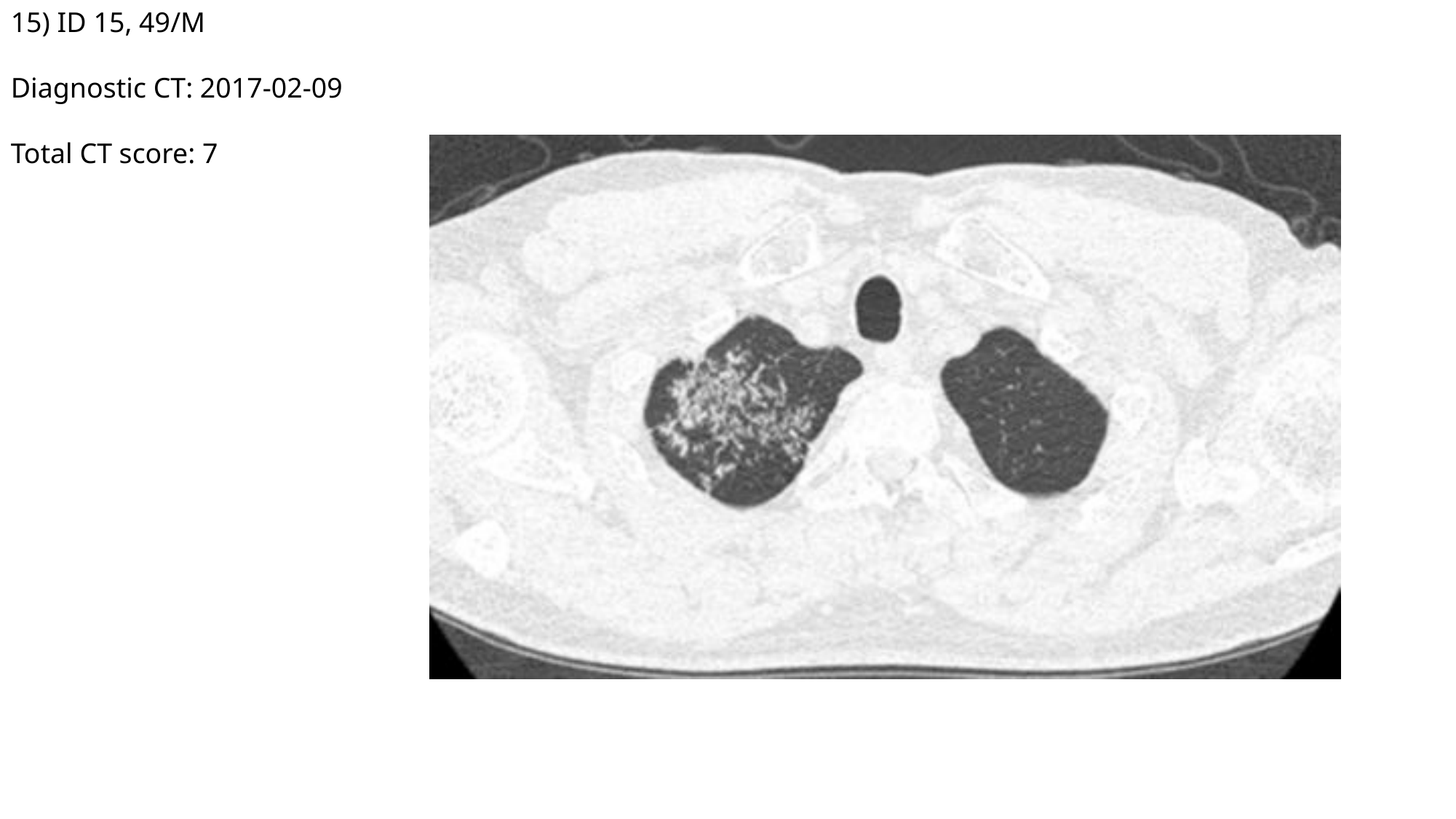

15) ID 15, 49/M
Diagnostic CT: 2017-02-09
Total CT score: 7

## Slide 17
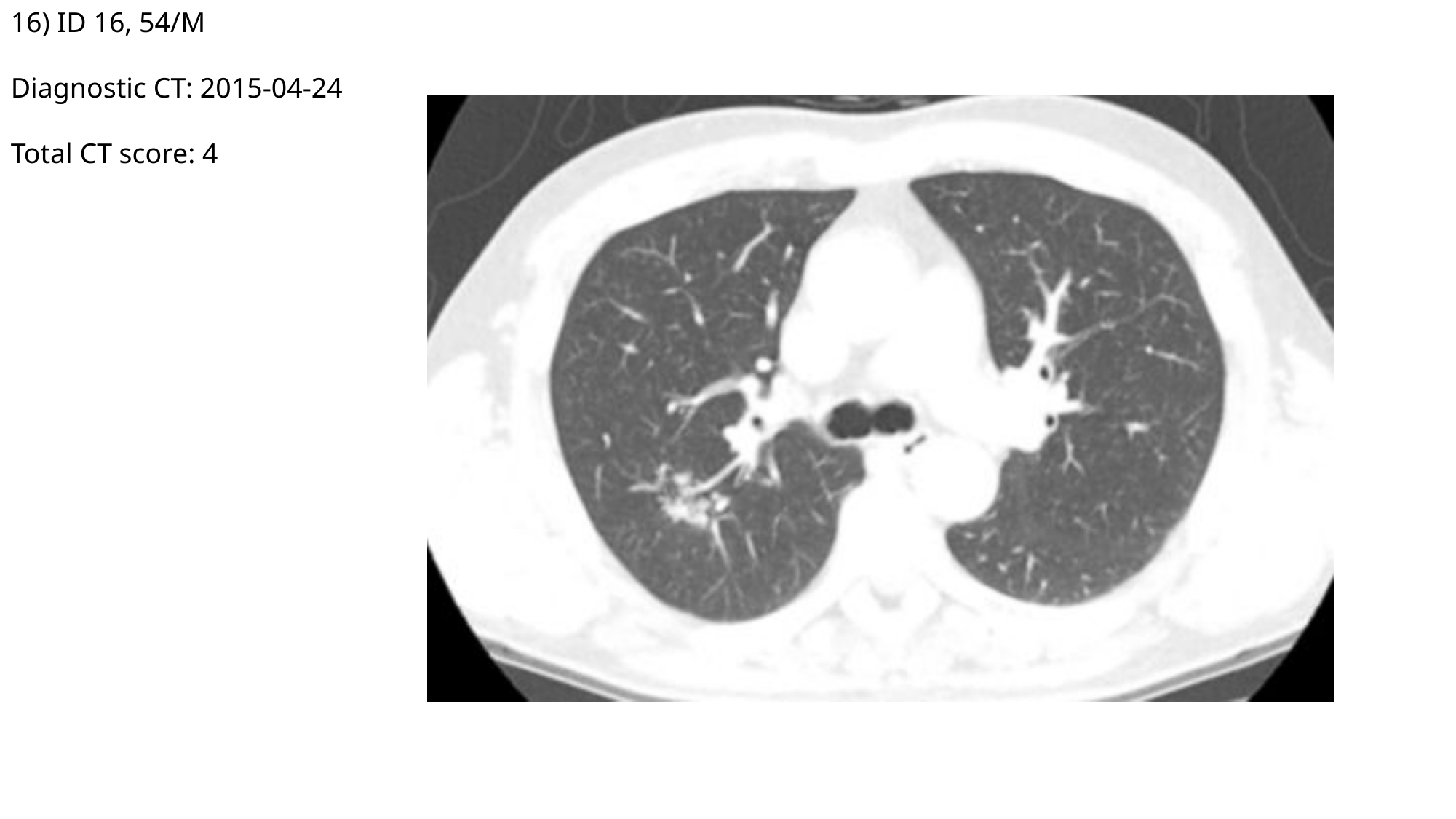

16) ID 16, 54/M
Diagnostic CT: 2015-04-24
Total CT score: 4

## Slide 18
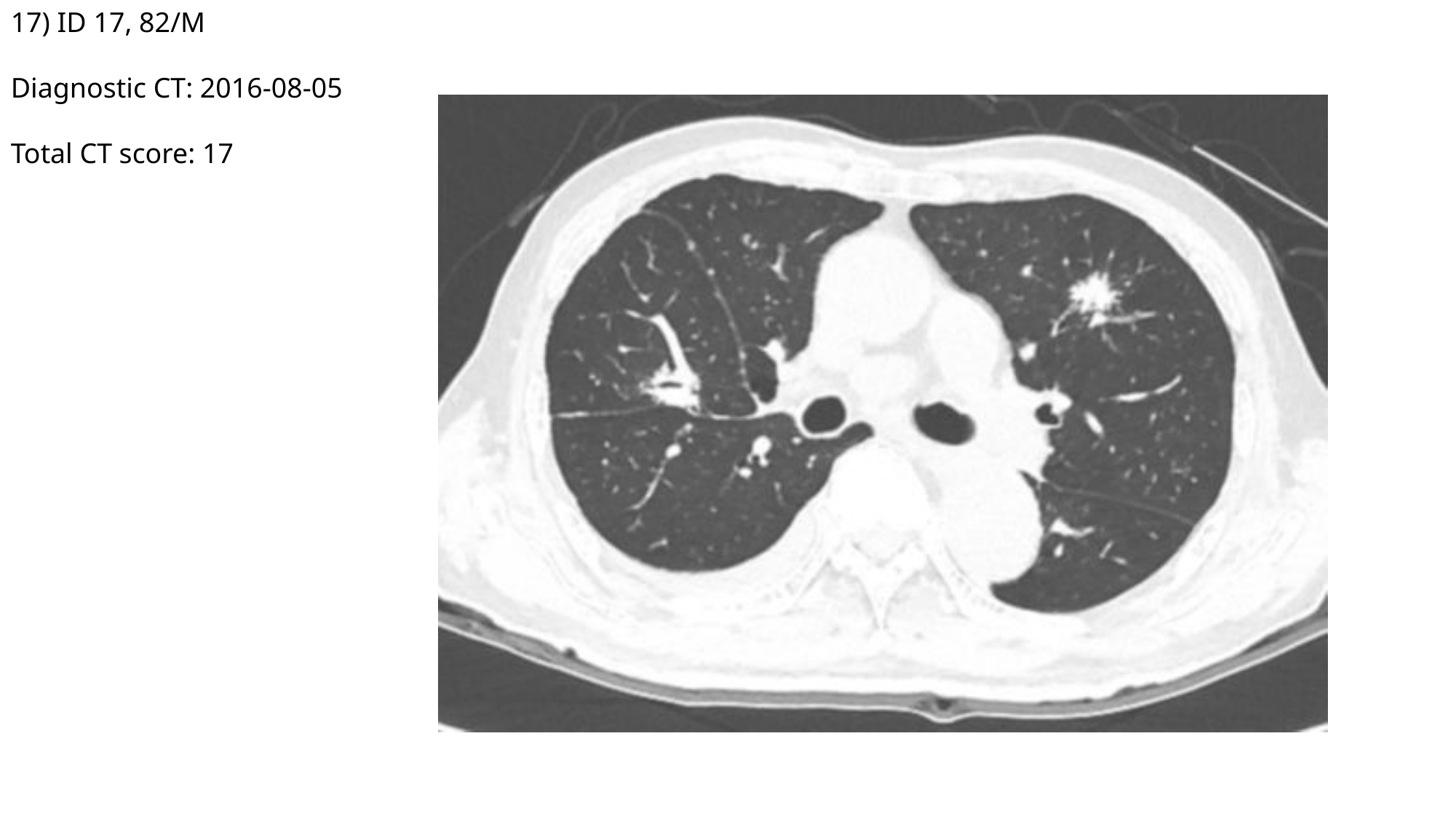

17) ID 17, 82/M
Diagnostic CT: 2016-08-05
Total CT score: 17

## Slide 19
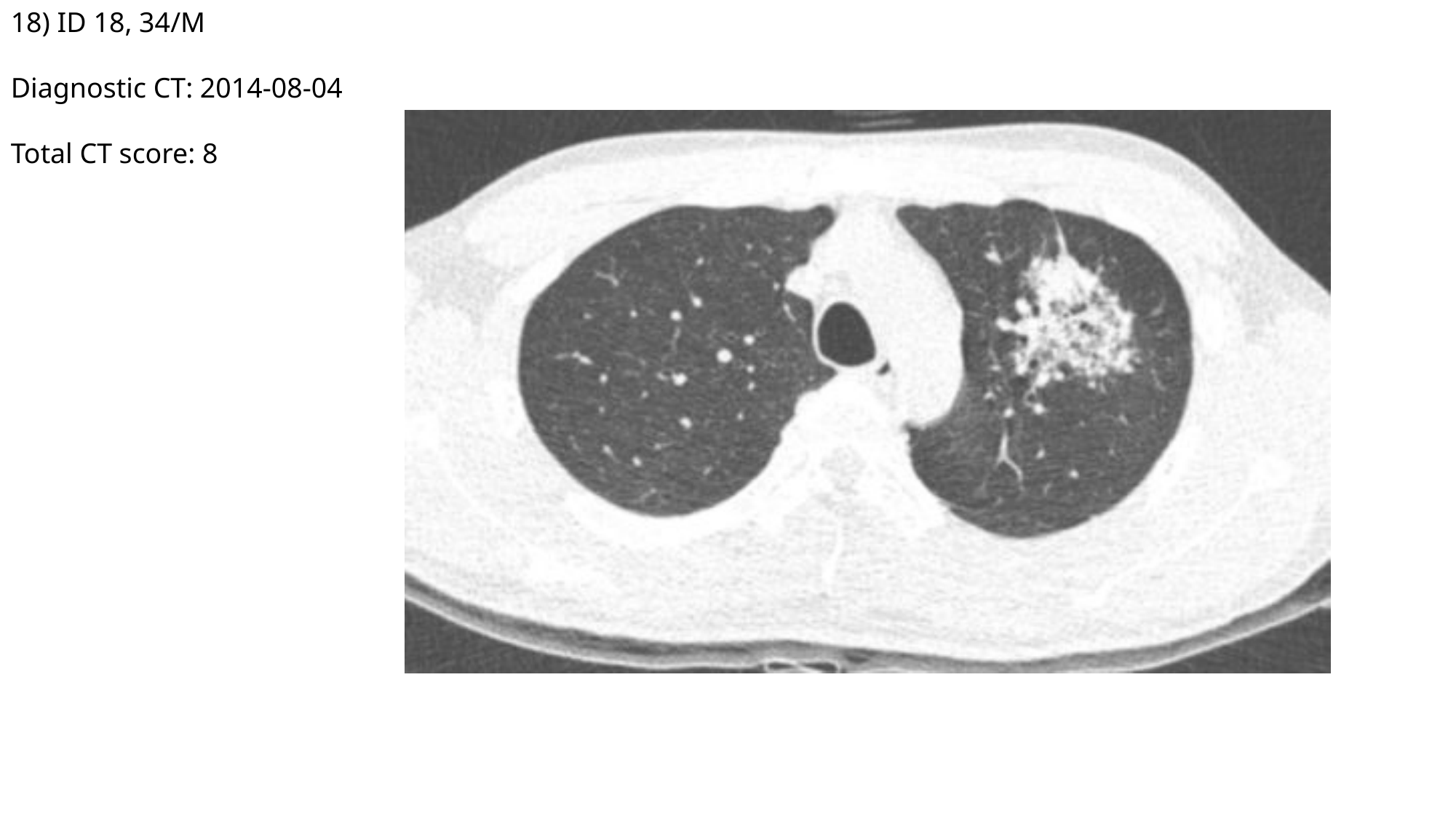

18) ID 18, 34/M
Diagnostic CT: 2014-08-04
Total CT score: 8

## Slide 20
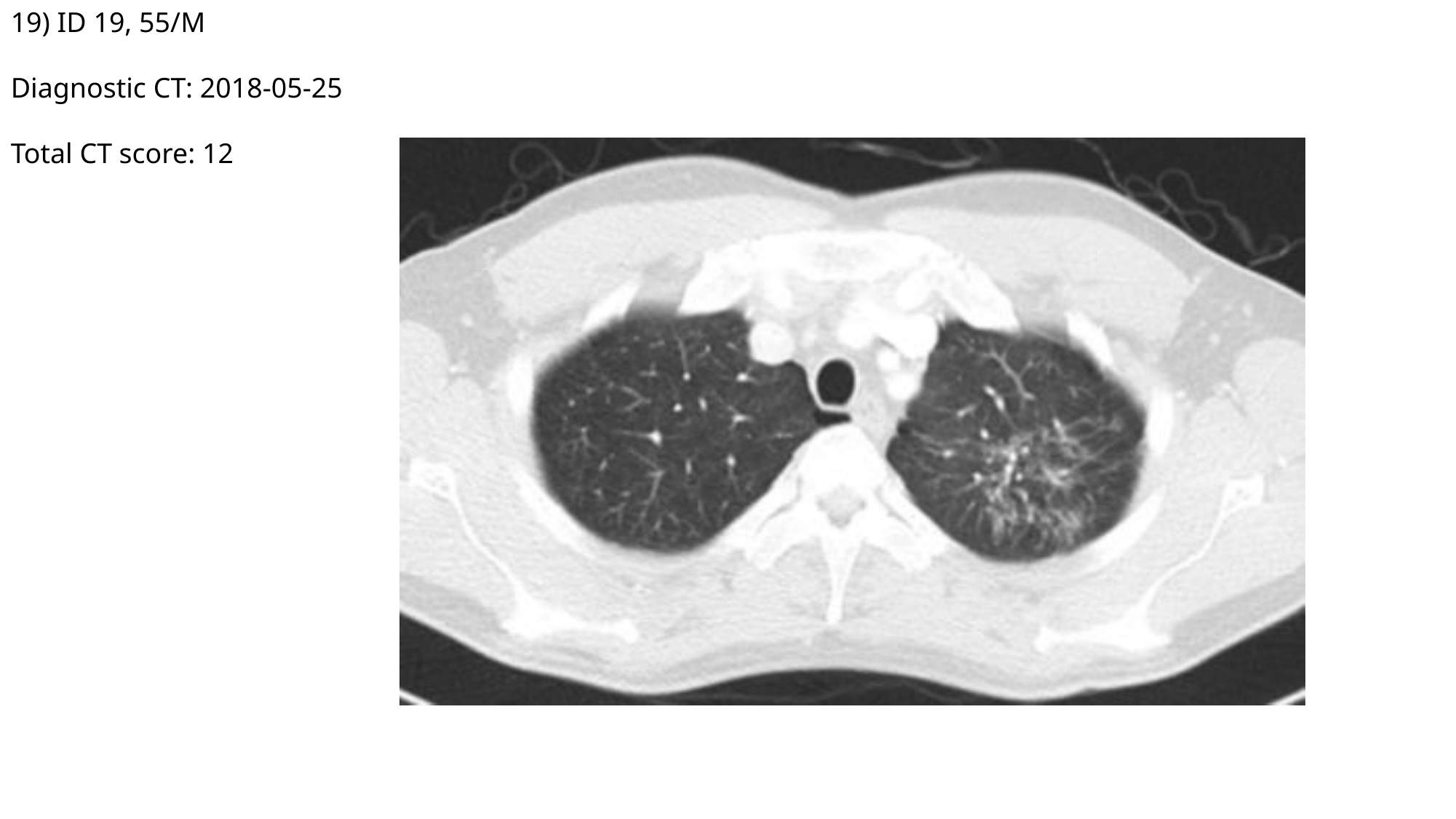

19) ID 19, 55/M
Diagnostic CT: 2018-05-25
Total CT score: 12

## Slide 21
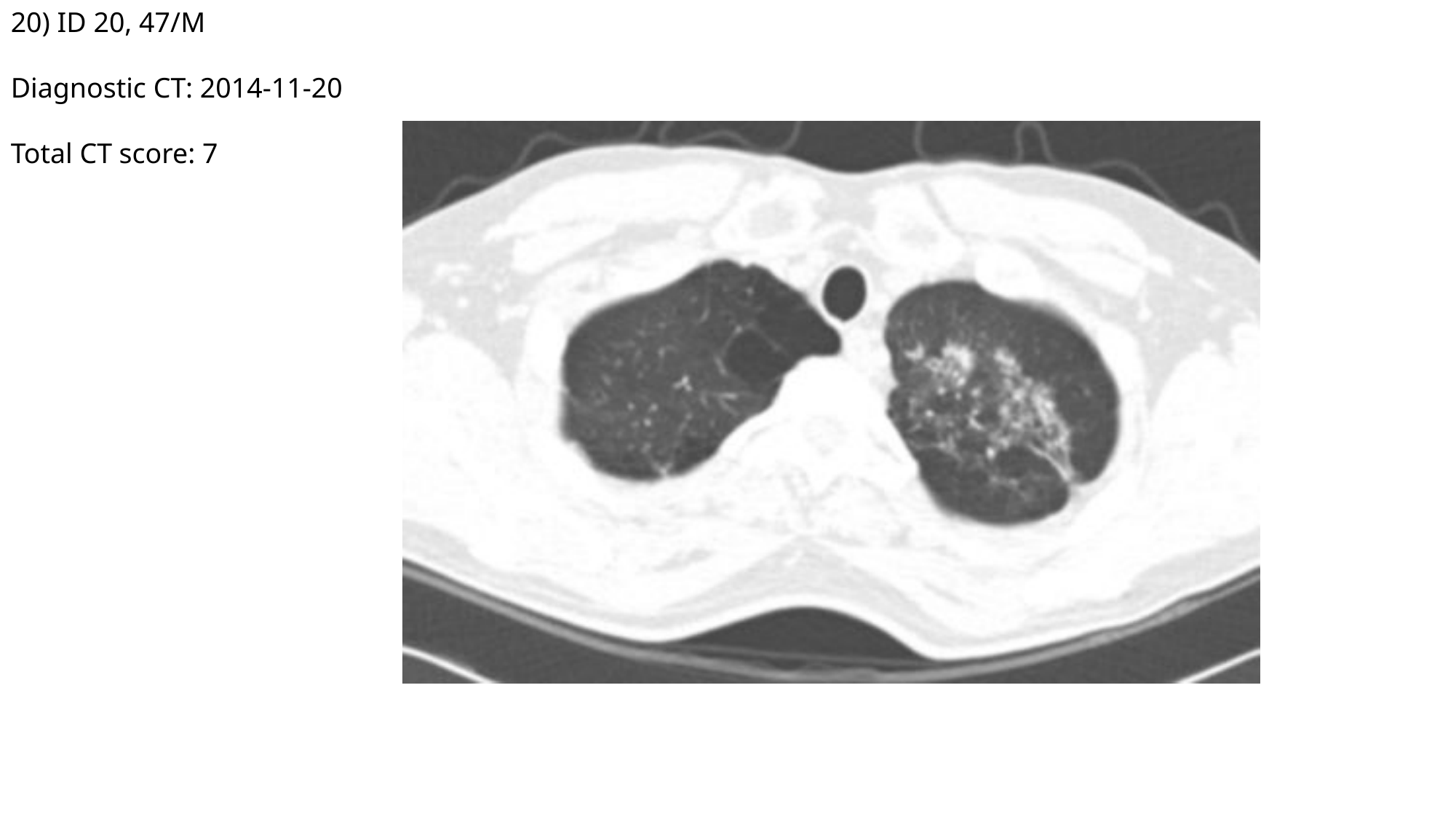

20) ID 20, 47/M
Diagnostic CT: 2014-11-20
Total CT score: 7

## Slide 22
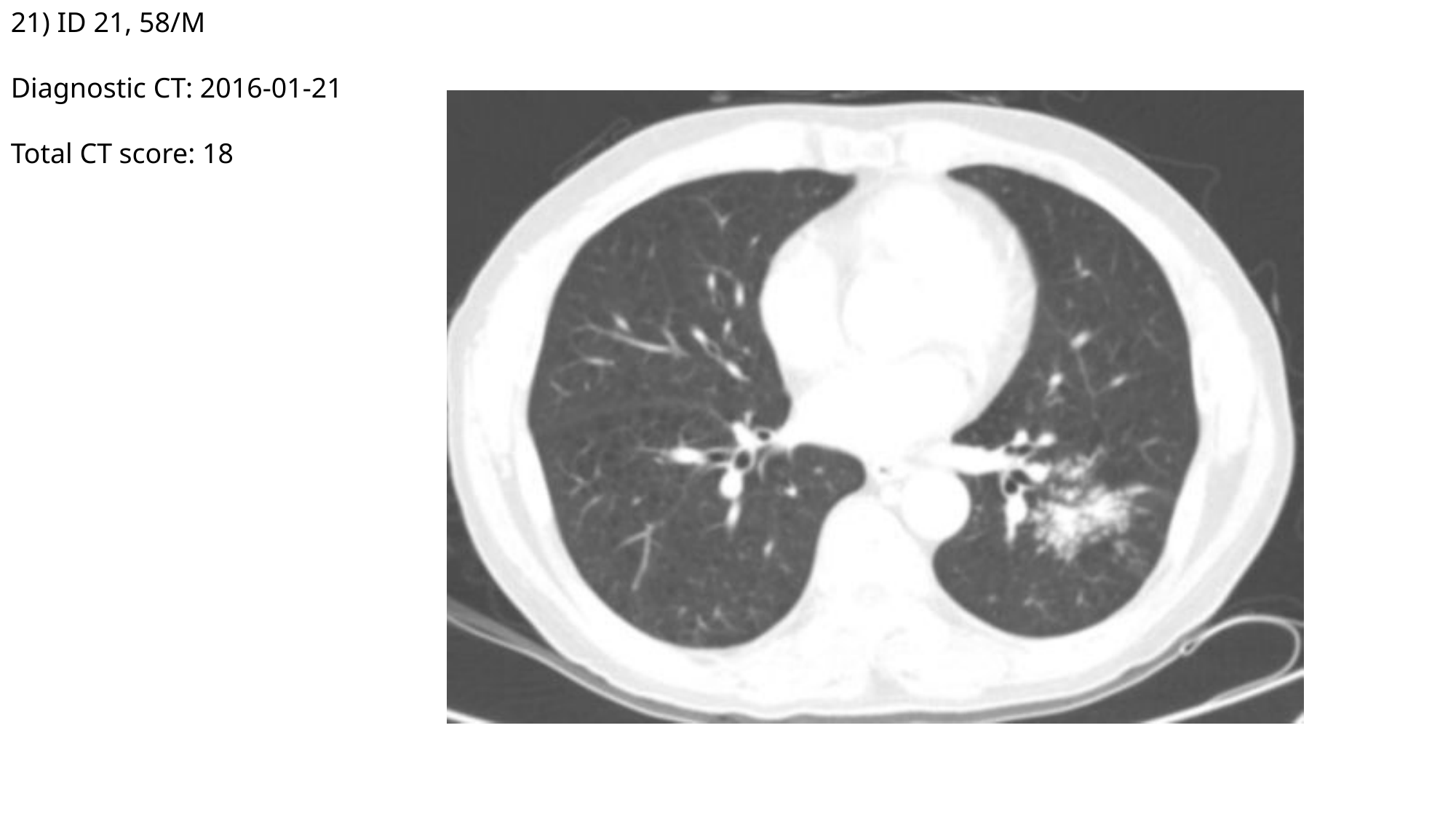

21) ID 21, 58/M
Diagnostic CT: 2016-01-21
Total CT score: 18

## Slide 23
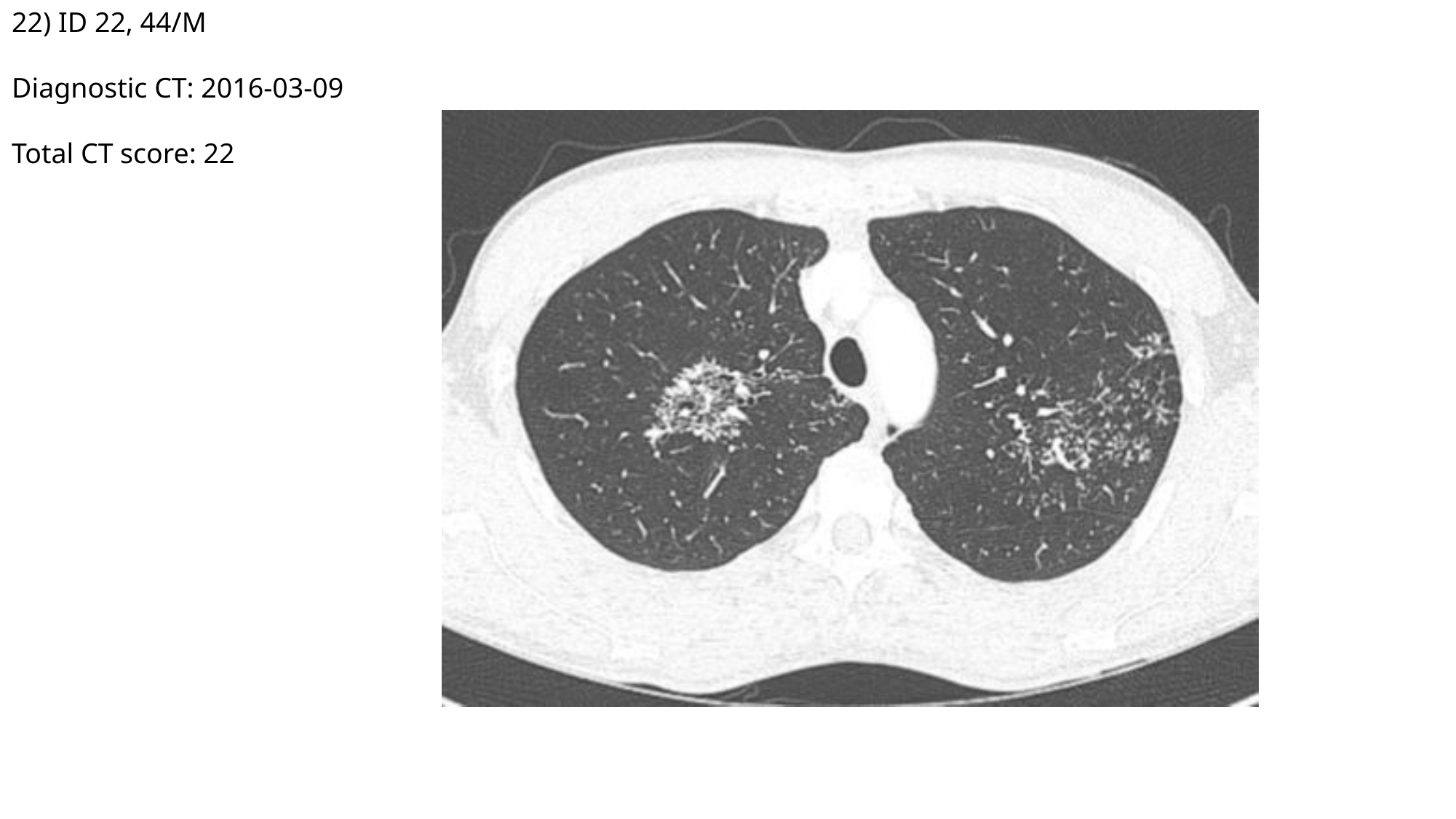

22) ID 22, 44/M
Diagnostic CT: 2016-03-09
Total CT score: 22
